# Presenilin2 D439A Mutation Induces Dysfunction of Mitochondrial Fusion/Fission Dynamics and Abnormal Regulation of GTPase Activity

**DOI:** 10.1007/s12035-023-03858-y

**Published:** 2023-12-30

**Authors:** Chenhao Gao, Junkui Shang, Zhengyu Sun, Mingrong Xia, Dandan Gao, Ruihua Sun, Wei Li, Fengyu Wang, Jiewen Zhang

**Affiliations:** 1 Department of Neurology, Zhengzhou University People’s Hospital, Henan Provincial People’s Hospital, Zhengzhou, 450003 Henan China; 2 Academy of Medical Sciences, Zhengzhou University, Zhengzhou, 450003 Henan China; 3 Department of Neurology, Henan University People’s Hospital, Henan Provincial People’s Hospital, Zhengzhou, 450003 Henan China

**Keywords:** Presenilin 2, Alzheimer’s disease, Transcriptome sequencing, Miro2, Mitochondrial dynamics

## Abstract

Alzheimer’s disease (AD) is an age-related progressive neurodegenerative disease, and approximately 10% of AD cases are early-onset familial AD (EOFAD), which is mainly linked to point mutations in genes encoding *presenilins* (*PS1* and *PS2*). Mutations in PS2 are extremely rare and have not received enough attention. Recently, studies have found that Rho GTPase activity is closely related to the pathogenesis of AD. In this study, we used transcriptome sequencing in PS2 siRNA-transfected SH-SY5Y cells and found a group of differentially expressed genes (DEGs) related to the regulation of GTPase activity. Among those DEGs, the most significantly downregulated was *Rho guanine nucleotide exchange factor 5* (*ARHGEF5*). GTPase activity in PS2 siRNA-transfected cells was significantly decreased. Then, we found that the expression of *ARHGEF5* and the GTPase activity of Mitochondrial Rho GTPase 2 (Miro2) in PS2 D439A mutant SH-SY5Y cells were significantly decreased. We found for the first time that PS2 can bind to Miro2, and the PS2 D439A mutation reduced the binding between PS2 and Miro2, reduced the expression of Miro2, and resulted in an imbalance in mitochondrial fusion/fission dynamics. In conclusion, *PS2* gene knockdown may participate in the pathogenesis of AD through the regulation of GTPase activity. The imbalance in mitochondrial dynamics mediated by the PS2 D439A mutation through regulation of the expression and GTPase activity of Miro2 may be a potential pathogenic mechanism of AD.

**Supplementary Information:**

The online version contains supplementary material available at 10.1007/s12035-023-03858-y.

## Introduction

Alzheimer’s disease (AD) is an age-related progressive neurodegenerative disease that mainly leads to cognitive impairment. Approximately 10% of AD cases occur in patients less than 65 years old and are called early-onset familial AD (EOFAD). The neuropathological changes in patients with AD are characterized by the deposition of extracellular amyloid-beta (Aβ) plaques and neurofibrillary tangles (NFTs) formed by intracellular phosphorylated tau protein [1]. The majority of familial AD (FAD) cases have been linked to point mutations in *Presenilin* genes, which encode two homologous proteins, presenilin 1 (PS1) and presenilin 2 (PS2) [2]. The best-characterized function of PS1 and PS2 is cleaving amyloid precursor protein (APP) to form Aβ as the catalytic component of the γ-secretase complex [3]. More than 300 mutations in *PS1* have been described worldwide, but mutations in *PS2* are extremely rare and have not received enough attention [4].

The complete function of PS2 and the mechanism by which it contributes to FAD pathogenesis are undetermined [4]. Currently, extensive studies have revealed that mitochondrial dysfunction exists independently and potentially lies upstream of Aβ deposition or NFT formation in AD pathogenesis [5, 6]. Mitochondria in AD brains showed fractured cristae, a reduced respiratory capacity, and increased fragmentation [7]. Mitochondria are dynamic organelles that constantly undergo fission, fusion and transport within cells [8]. Mitochondrial Rho GTPase (Miro) proteins belong to a family of evolutionarily conserved atypical GTPases [9], and each of the two homologs, Miro1 and Miro2, contain two GTPase domains flanking a pair of EF-hand motifs and a C-terminal transmembrane domain that anchors the protein to the outer mitochondrial membrane (OMM) [10]. The functions of Miro1/2 include maintaining mitochondrial morphogenesis, mitochondria–endoplasmic reticulum (ER) communication, and apoptosis [11]. Miro1/2 can also regulate mitochondrial morphology and fission/fusion by interacting with mitofusin1 (Mfn1) and mitofusin2 (Mfn2) [11]. Structure‒function studies have indicated that the GTPase domains play a key role in regulating mitochondrial morphology by regulating mitochondrial fusion and fission [12].

A previous study found that PS2 and PS1 can interact with the small GTPase Rab11, and the interaction domain was mapped to the C-terminal end of PS1 [13]. PS1 can also interact with Rac1, which belongs to the Rho family of small G proteins [14]. However, there is no relevant study on whether PS2 can interact with Miro proteins. The PS2 D439A mutation was first reported in 2001 in an early-onset AD patient with a missense mutation in exon 12 C.439 A > C of the *PS2* gene, resulting in the substitution of the aspartic acid encoded by the 439 codon at the C-terminus of PS2 with alanine [15]. A previous study evaluated the effect of the PS2 D439A mutation on Aβ levels when this PS2 mutant was co-expressed with APP carrying the Swedish mutation (APPsw) in PS^1/2−/−^ mouse embryonic fibroblasts (MEFs); the PS2 D439A mutation did not increase either the Aβ42 level or the Aβ42/40 ratio [16]. Polymorphism phenotype v2 (PolyPhen-2) software was used to evaluate the pathogenicity of the variant PS2 D439A, the result (score 0.961) showed that this mutation is probably damaging, and Sorting Intolerant From Tolerant (SIFT) was used to predict that an aspartate-to-alanine substitution in the PS2 D439A mutation may disrupt the encoded proteins [17]. However, few studies have conducted relevant experiments to verify the pathogenic mechanism of the PS2 D439A mutation.

In this study, we first conducted transcriptome sequencing analysis of SH-SY5Y cells after *PS2* gene knockdown and then performed Gene Ontology (GO)/Kyoto Encyclopedia of Genes and Genomes (KEGG) and Disease Ontology (DO) enrichment analyses to screen the differentially expressed genes (DEGs) possibly involved in the regulation of mitochondrial dynamics and the pathogenesis of AD. Next, the relevant DEGs and their functions were verified in PS2 D439A cells, and the changes in the expression of proteins related to mitochondrial dynamics were verified to further explore the potential mechanism by which the PS2 D439A mutation leads to AD and investigate its correlation with imbalanced mitochondrial dynamics.

## Materials and Methods

### Cell Culture

Human embryonic kidney 293 T cells were a gift from Professor Fengmin Shao and maintained in Dulbecco’s modified Eagle’s medium (DMEM) (HyClone, USA) supplemented with 10% fetal bovine serum (FBS; Gibco, USA). Lentiviruses were produced in human embryonic kidney 293 T cells. The human neuroblastoma clonal SH-SY5Y cell line was purchased from the Cell Resources Center of Shanghai Institute of Life Science, Chinese Academy of Sciences (Shanghai, China), and was grown in DMEM/F-12 (HyClone, USA) supplemented with 10% FBS (Gibco BRL, USA). All culture media contained 100 IU/ml penicillin and 100 mg/ml streptomycin (Gibco, USA). All cells were cultured at 37 °C in a humidified atmosphere of air containing 5% CO_2_.

### Construction of Expression Plasmids

The interference vector pLKD-CMV-Puro-U6-shRNA was purchased from OBiO Technology (Shanghai) Co., Ltd. After enzymatic digestion with AgeI and EcoRI, three siRNA targets were designed, with the sequences CCCTCAAATACGGAGCGAA, CCATCAAGTCTGTGCGCTT, and GCCTCTGAGAATGCTGGTA. The sequence of the designed nontargeting negative control (scramble) was TTCTCCGAACGTGTCACGT. The full-length human *PS2* cDNA sequence (NCBI accession no. NM_000447) was amplified from total genomic DNA extracted from SH-SY5Y cells by PCR using the forward primer 5′-gaggcgcgccgccaccATGCTCACATTCATGGCCTCTG-3′ and reverse primer 5′-tggtggccacgtggatgtTCAGATGTAGAGCTGATGGGAGG-3′. The lentiviral expression vector pLV-EF1a-CFP-CMV-Puro-WPRE was purchased from BrainVTA Co., Ltd. (Wuhan, China). Human PS2 cDNA was inserted into pLV-EF1a-CFP-CMV-Puro-WPRE, and Phanta Max Super-Fidelity DNA Polymerase (Vazyme Biotech, China) was used to construct the pLV-EF1a-PS2 wild-type (WT)-CFP-CMV-Puro-WPRE expression vector. Positive clones were identified by colony PCR and double digestion, and Sanger sequencing was then performed for further confirmation. The PS2 D439A mutation was introduced into the pLV-EF1a-PS2 WT-CFP-CMV-Puro-WPRE expression vector by homologous recombination with primers (forward primer: 5′-tcgtgatctagaGGCGCGCCgccaccatgctcacattcatgg-3′, reverse primer: 5′-ttgctcatggtggccacgtggatgtagagctgatgggaggccagggtgGccatgaacggccgcac-3′) designed to target both ends of the mutated sequence. Both the PS2 WT and PS2 D439A mutant cDNA sequences were verified by direct sequencing.

### Lentivirus Packaging and Infection

293 T cells plated in 15 cm dishes were cotransfected with 20 µg of the recombinant PS2 lentiviral expression vectors, 15 µg of pHelper1.0, and 10 µg of pHelper2.0 using Lipofectamine 3000 (Invitrogen, Carlsbad, CA). Six hours (h) after transfection, the culture medium was refreshed. Virus-containing supernatants were collected at 48 h and 72 h after transfection, centrifuged at 3500 rpm for 10 min, filtered with a 0.45-µm sterile filter, and transferred into an ultracentrifuge tube, which was placed into an ultracentrifuge, and centrifuged at 30,000 rpm and 4 ℃ for 2 h. The samples were then filtered through a low protein-binding filter (0.22 µm), aliquoted, and stored at − 80 ℃. Then, 150 µl of precooled Dulbecco’s phosphate-buffered saline (DPBS) was added to each centrifuge tube, and the precipitate was resuspended, transferred into a 1.5-ml sterile EP tube, and allowed to stand overnight at 4 ℃. The viral suspension was collected, centrifuged at 6000 rpm and 4 ℃ for 5 min, filtered through a 0.22-µm sterile filter, and transferred to a new sterile EP tube. After subpackaging, the lentivirus was stored in a − 80 ℃ freezer.

When the SH-SY5Y cells reached 20–30% confluence in 6-well plates, the lentiviral supernatant was added to each well to achieve a multiplicity of infection (MOI) of 20. For viral infection, the cells were incubated for 2 h with the lentiviral supernatant in 1 ml of serum-free medium, and an equal volume of DMEM/F-12 supplemented with 10% FBS was added. The medium was replaced with fresh medium 24 h after infection. Selection was initiated 72 h after infection by adding 2 µg/ml puromycin (Sigma-Aldrich, USA). Recombinant cell lines were further subcultured in culture medium containing 1 µg/ml puromycin, and the expression profiles of the cells expressing PS2 siRNA, PS2 WT, and the PS2 D439A mutant were monitored by quantitative real-time PCR (qRT‒PCR) and Western blot analysis.

### Reverse Transcription and qRT-PCR Analysis

Total RNA was extracted with an RNAsimple Total RNA Kit (DP419, Tiangen, China) according to the manufacturer’s instructions. Reverse transcription was performed using PrimeScript™ RT Master Mix (RR036A, TaKaRa, USA). qRT-PCR was performed utilizing TB Green® Premix Ex Taq™ II (RR820A, TaKaRa, USA) on a PCR instrument (7500 Real-Time PCR Detection System, Applied Biosystems, USA). Target cDNA expression was measured using the relative quantification method. Specific primers (see Table 1) were used for cDNA amplification. The thermal cycling conditions used for qRT‒PCR were as follows: 95 °C for 30 s, followed by 40 cycles at 95 °C for 5 s and 60 °C for 34 s.

**Table 1:** Primers of qRT-PCR

Gene	Primer direction	Sequence (5′–3′)
PS2	Forward	TGCGCTCATGGCCCTAGTGTTCA
	Reverse	TGGGCTCATTTCTCTCCTGGGCAGTT
ARHGEF5	Forward	CAAACCTGCCATCTACAGCTC
	Reverse	AAGAACCGTAGGCGGGAGA
GNG3	Forward	CCCCGGTGAACAGCACTATG
	Reverse	GGCATCACAGTAAGTCATCAGG
JUN	Forward	TCCAAGTGCCGAAAAAGGAAG
	Reverse	CGAGTTCTGAGCTTTCAAGGT
RAPGEF5	Forward	TCTTGTGCGTCTAACATCTGC
	Reverse	GAATCTGGAACACTTTCGGCTT
SFRP1	Forward	ACGTGGGCTACAAGAAGATGG
	Reverse	CAGCGACACGGGTAGATGG
PLXNA4	Forward	GTCATTTGTCACATTCCGAGGA
	Reverse	GCTTGTAAATCCGATTGACGGC
KIAA1614	Forward	GGCTCCTCGTACCCAAAACC
	Reverse	TGTCATGGTCAGGAAGTGTCC
RELN	Forward	CAACCCCACCTACTACGTTCC
	Reverse	TCACCAGCAAGCCGTCAAAAA
SPRY1	Forward	GCAGTGGCAGTTCGTTAGTTG
	Reverse	CAGTAGGCTGAATCTCTCTCTCA
RGPD8	Forward	GATATTAGGGGCCGGAAGAAGG
	Reverse	AGGGGCTTCAGGTTCATTGTAG
β-actin	Forward	ATCATGTTTGAGACCTTCAACA
	Reverse	CATCTCTTGCTCGAAGTCCA
GAPDH	Forward	GCAAATTCCATGGCACCGTCAAGG
	Reverse	CGCCAGCATCGCCCCACTTG

### Western Blot

Cells were lysed in Western and IP lysis buffer (Beyotime, China) supplemented with an EDTA-free complete protease inhibitor cocktail tablet (Thermo, USA) on ice for 30 min. The lysate was centrifuged at 13,000 rpm for 15 min at 4 °C, and then, the supernatant was transferred to a fresh tube and stored at − 80 °C. Protein concentrations were determined using a bicinchoninic acid (BCA) assay (Thermo, USA). Proteins in the samples were separated by 8–12% sodium dodecyl sulfate–polyacrylamide gel electrophoresis (SDS‒PAGE) and transferred to 0.45-µm PVDF Transfer Membranes (Millipore, USA). After blocking in 5% nonfat milk, the membranes were incubated with a primary antibody (4 °C, overnight) prior to washing and incubation with horseradish peroxidase (HRP)–conjugated secondary antibodies (1 h, room temperature). The following primary antibodies were used: rabbit anti-PS2 (Abcam, Cat #ab51249, 1:1000), rabbit anti-βActin (Beyotime, Cat #AF5003, 1:2000), rabbit anti-Miro1 (ABclonal, Cat #A5838, 1:1000), rabbit anti-Miro2 (Proteintech, Cat #11,237–1-AP, 1:2000), rabbit anti-Mfn1 (Proteintech, Cat #13,798–1-AP, 1:1000), rabbit anti-Mfn2 (Proteintech, Cat #12,186–1-AP, 1:1000), rabbit anti-Drp1 (Proteintech, Cat #12,957–1-AP, 1:1000), rabbit anti-Cyt c (Proteintech, Cat #10993–1-AP, 1:1000), rabbit anti-BCL-2 (Proteintech, Cat #12789–1-AP, 1:1000), rabbit anti-BAX (Proteintech, Cat #50599–2-Ig, 1:2000), and rabbit anti-VDAC1 (Proteintech, Cat #55259–1-AP, 1:1000). The secondary antibodies used were HRP-labeled goat anti-rabbit IgG (H + L) (Beyotime, Cat #A0208, 1:1500) and HRP-labeled goat anti-mouse IgG (H + L) (Beyotime, Cat #A0216, 1:1500). After a final wash step, immunodetection was performed using enhanced chemiluminescence (Immobilon™ Western Chemiluminescent HRP Substrate (ECL), Millipore, USA); immunoreactive protein bands were digitally imaged and band densities were quantified with UVP VisionWorks Acquisition/Analysis Software (Analytik Jena, USA).

### Mitochondrial Protein Extraction

A Qproteome Mitochondrial Isolation Kit (Qiagen, Germany) was used in this experiment. SH-SY5Y cells (1 × 10^7^) were harvested and centrifuged at 500 × g for 10 min at 4 °C and then washed using 0.9% sodium chloride solution. The cell pellet was resuspended in 1 ml of ice-cold lysis buffer containing protease inhibitor solution and incubated for 10 min at 4 °C on an end-over-end shaker. Next, each sample was centrifuged at 1,000 × g at 4 °C for 10 min, and the supernatant containing the cytosolic proteins was collected in a clear 1.5-ml tube for further analysis. The cell pellet was resuspended in 1.5 ml ice-cold disruption buffer containing protease inhibitor solution. Then, the lysate was drawn into a syringe using a blunt needle and ejected; this step was repeated 10 times. The lysate was centrifuged again at 1000 × g and 4 °C for 10 min, and the supernatant was transferred to a new tube and centrifuged at 6000 × g for 10 min at 4 °C, and the resulting supernatant was removed. The pellet containing the mitochondrial fraction was washed with 1 ml of Mitochondria Storage Buffer, centrifuged at 6000 × g for 20 min at 4 °C, and resuspended in Mitochondria Storage Buffer for further analysis.

### Transcriptome Sequencing

The specific steps were as follows: preparation of PS2 siRNA knockdown cell line samples infected by lentiviral transduction, RNA extraction and detection, library construction, quality inspection, sequencing, bioinformatics analysis, etc. The libraries were sequenced using an Illumina HiSeq™ 4000 instrument by Gene Denovo Biotechnology Co., Ltd. (Guangzhou, China). The raw RNA sequencing (RNA-seq) reads were mapped to the reference genome using HISAT2 (version 2.1.0) [18]. With StringTie software, a fragment per kilobase of transcript per million mapped reads (FPKM) value was calculated for each transcription region to quantify its expression abundance and variations. DEGs were identified using DESeq2 [19] based on the parameters of false discovery rate (FDR) value < 0.05 and |log_2_ fold change(FC)|> 1. Raw RNA sequencing data were deposited in the NCBI Sequence Read Archive database (PRJNA963881).

All DEGs were mapped to GO terms in the GO database (http://www.geneontology.org/), gene numbers were calculated for each term, and hypergeometric tests were used to identify significantly enriched GO terms in DEGs compared to the genome background. The calculated *p*-value underwent FDR correction, with a threshold of FDR ≤ 0.05. GO terms that met this condition were defined as significantly enriched GO terms in DEGs. KEGG is a leading public pathway-related database, DO is a database (http://disease-ontology.org/) that describes gene functions associated with diseases, and KEGG and DO analysis methods are identical to those used in GO analysis.

### Detection of GTPase Activity

A GTPase assay kit (ab270553, Abcam, USA) was used to detect the changes in GTPase activity in SH-SY5Y cells infected with various lentiviruses. The kit includes a GTP reaction substrate and provides a simple and direct method to measure GTPase activity in the sample to be tested. GTPases catalyze the hydrolysis of GTP to GDP and free phosphate (Pi). The PiColorLock™ reagent in the kit and the Pi released during the enzymatic reaction form a dark green Pi-dye complex, and its color depth is directly proportional to the enzyme activity. After reaction for 30 min, the absorbance value was measured at 590–660 nm. All test steps were carried out according to the instructions. As directed, no reagent in contact with the sample to be tested contained phosphate.

### Co-immunoprecipitation (Co-IP)

Cells were lysed in Western and IP Lysis Buffer (Beyotime, China) supplemented with an EDTA-free complete protease inhibitor cocktail tablet (Thermo, USA) on ice for 30 min, the lysates were centrifuged (13,000 rpm, 15 min, 4 °C), and the total protein in the supernatant was quantified. Then, equal amounts of protein (300 ~ 500 μg) were added to 20 μl of Pure Proteome Protein A/G Mix Magnetic Beads (Millipore, USA) and incubated with 1 μg of normal rabbit IgG (Beyotime, Cat #A7016) for 2 h at 4 °C. Then, the beads were incubated with 1 μg of a rabbit anti-PS2 (Abcam, Cat #ab51249), rabbit anti-Miro1 (ABclonal, Cat #A5838), or rabbit anti-Miro2 (Proteintech, Cat #11,237–1-AP) antibody overnight at 4 °C under constant rotation. Then, 25 μl of beads was added to each pretreated protein sample, and the mixture was incubated for 30 min at room temperature with constant rotation. The immune complexes were washed three times with 500 µl of PBST, denatured in 60 μl of sample buffer and analyzed by western blotting. Total lysate (20 µg) was used as input. Each experiment was performed at least three times.

### GST Pull Downs

The cDNAs encoding amino acids 1–87 and amino acids 271–361 of PS2 were ligated into the BamHI/XhoI sites of the plasmid pGEX-4 T-3, and then expressed as a glutathione S-transferase(GST)-tagged fusion protein transformed in *E. coli* BL21. The cDNAs encoding amino acids 1–219 and 220–592 of Miro2 were inserted into the BamHI/XhoI sites of the plasmid pET-32a, and then expressed as a HIS-tagged fusion protein transformed in *E. coli* BL21. Recombinant GST fusion proteins and recombinant HIS fusion proteins were induced using 0.1 mM isopropyl β-D-thiogalactoside (IPTG) for 2 h at 30 °C and extracted and purified as previously described [20]. Bound material was eluted with 4 × SDS sample buffer and processed for SDS-PAGE and Western blot.

### Synthesis of Miro2 Adenovirus (Ad-Miro2) 

Ad-Miro2 and the corresponding normal control adenovirus (Ad-NC) were constructed and synthesized by OBiO Technology (Shanghai) Co., Ltd. Using the vector pAdeno-MCMV-MCS-Myc, the CDS of Miro2 was amplified by PCR, and the pAdeno-MCMV-MCS-Myc-Miro2 overexpression vector was then constructed. The overexpression efficiency of Ad-Miro2 was verified by Western blotting.

### Mitochondrial Transmembrane Potential Measurements in Living Cells

A mitochondrial membrane potential (MMP) assay kit with JC-1 (Beyotime, China) was used in this assay. SH-SY5Y cells (2 × 10^5^ cells/well) were seeded in six-well plates. For one well of the six-well plate, the culture medium was removed, and the cells were washed once with PBS or another appropriate solution according to the specific experiment. Then, 1 ml of cell culture medium and 1 ml JC-1 dyeing solution were added and mixed well. The cells were incubated at 37 ℃ for 30 min. During incubation, every 1 ml of JC-1 staining buffer (5 ×) was added to 4 ml distilled water to prepare JC-1 staining buffer (1 ×), which was placed in an ice bath. After incubation at 37 ℃, the supernatant was removed and washed twice with JC-1 staining buffer (1 ×), and then, 2 ml of cell culture medium was added. The changes in MMP were observed by fluorescence microscopy. The results of JC-1 measurement were expressed as the ratio of green/red mass fluorescent cells detected at 490 nm and 525 nm excitation. Samples were analyzed using a fluorescence microscope (BX53, Olympus, Japan). Images were exported as TIFF, background subtracted and analyzed with Image-Pro plus 6.0 software (Media Cybernetics, Rockville, MD, USA).

### Transmission Electron Microscopy (TEM) Analysis

In brief, the culture medium of each group of cells was discarded, and 2.5% glutaraldehyde solution was added to fix the cells for 5 min at room temperature. Then, the cells were gently harvested by scraping in one direction with a cell scraper. The cell suspension was aspirated and transferred to a centrifuge tube with a pasteurized pipette and centrifuged at 2000 rpm for 2 min. The ideal size of the cell mass was similar to that of a mung bean. After discarding the fixation solution, new 2.5% glutaraldehyde solution was added, and the cell mass was gently picked up and suspended in the fixation solution for 1 h. Then, the cells were dehydrated in an ethanol dilution series and embedded in epoxy embedding medium (Sigma-Aldrich, USA). Ultrathin sections were sliced at a 60-nm thickness and observed under an electron microscope (Hitachi TEM system, Japan).

### Reactive Oxygen Species Analysis

A ROS assay kit (Beyotime, China) was used to detect ROS using the fluorescent probe 2’,7’-dichlorodihydrofluorescein diacetate (DCFH-DA). DCFH-DA itself has no fluorescence and can freely pass through the cell membrane. After entering the cell, it can be hydrolyzed by esterases in the cell to produce DCFH. ROS in cells can oxidize nonfluorescent DCFH to produce fluorescent DCF. The level of ROS in cells can be determined by detecting the fluorescence of DCF. Samples were analyzed using a fluorescence microscope (BX53, Olympus, Japan). Images were exported as TIFF files, subjected to background subtraction and analyzed with Image-Pro Plus 6.0 software (Media Cybernetics, Rockville, MD, USA).

### Enzyme-Linked Immunosorbent Assay (ELISA)

Levels of oxidative stress–related factors were measured using a malondialdehyde (MDA) ELISA kit (Elabscience, E-EL-0060c, China), human superoxide dismutase 2 (SOD2) ELISA kit (Elabscience, E-EL-H6188, China), and glutathione peroxidase (GSH-Px) ELISA kit (MEIMIAN, MM-0457H2, China), following the protocols of the manufacturer. Briefly, cells were washed twice with PBS, and harvested with 0.25% trypsin. The cell suspension was centrifuged at 1000 × g for 5 min, and 1 × 10^6^ cells were resuspended in 150 μl of cold PBS. The freeze–thaw process was repeated several times until the cells were fully lysed. After centrifugation for 10 min at 1500 × g at 4 °C, the supernatant was collected for assay.

### ATP Measurement

ATP contents in SH-SY5Y cells were analyzed using an ATP Chemiluminescence Assay Kit (Elabscience, E-BC-F002, China) according to the manufacturer’s protocol. Briefly, cells were resuspended in extracting solution and incubated in a boiling water bath for 10 min; after cooled, the samples were centrifuged at 10,000 × g for 10 min at 4 °C. The supernatant was collected for further assays. Chemiluminescent signals were detected by Multifunctional Microplate Reader Synergy H1 (BioTek, USA).

### Cell Viability and Cell Apoptosis Analysis

A cell counting kit-8 (CCK8) assay kit (Dojindo, Japan) was used to assess cell viability. Cells (6 × 10^3^ cells/well) were seeded in 96-well plates and cultured for 24 h. Then, the culture supernatant was removed, 10 μl of CCK8 solution in 100 µl of culture medium was added per well, and the cells were incubated in 5% CO_2_ at 37 °C for 4 h. The absorbance at 450 nm was measured with a microplate spectrophotometer (Synergy H1, BioTek Instruments, USA).

The Aβ25-35 peptide (MedChemExpress, USA) was dissolved in sterilized deionized water to a concentration of 1 mM and stored at − 80 ℃ until use. To prepare aggregated Aβ25-35 fibrils, Aβ25-35 peptide was placed in a 37 ℃ incubator for 7 days. Then, the Aβ25-35 fibril solutions were diluted to 40 µM with serum-free DMEM-F12 and added to the cultured cells for incubation in an incubator containing 5% CO_2_ at 37 °C for 48 h. Apoptotic cells were detected by staining with the nuclear dye Hoechst 33,342 (Invitrogen, USA), which reveals fragmented or intensely stained nuclei. Nuclear morphology was visualized under a fluorescence microscope (BX53, Olympus, Japan). Images were exported as TIFF files, subjected to background subtraction and analyzed with Image-Pro Plus 6.0 software (Media Cybernetics, Rockville, MD, USA).

### Statistical Analysis

Statistical analysis was performed and graphs generated with GraphPad Prism 6.0 software. Data normality was assessed using the Shapiro–Wilk test. Data are expressed as the means ± SDs. Two-tailed Student’s *t* test and either one-way ANOVA followed by Dunnett’s post hoc test or two-way ANOVA were used to analyze individual differences between two groups and among more than two groups, respectively. *P* < 0.05 was considered to indicate a statistically significant difference.

## Results

### Screening for the Best Interference Sequence

**Fig. 1:**
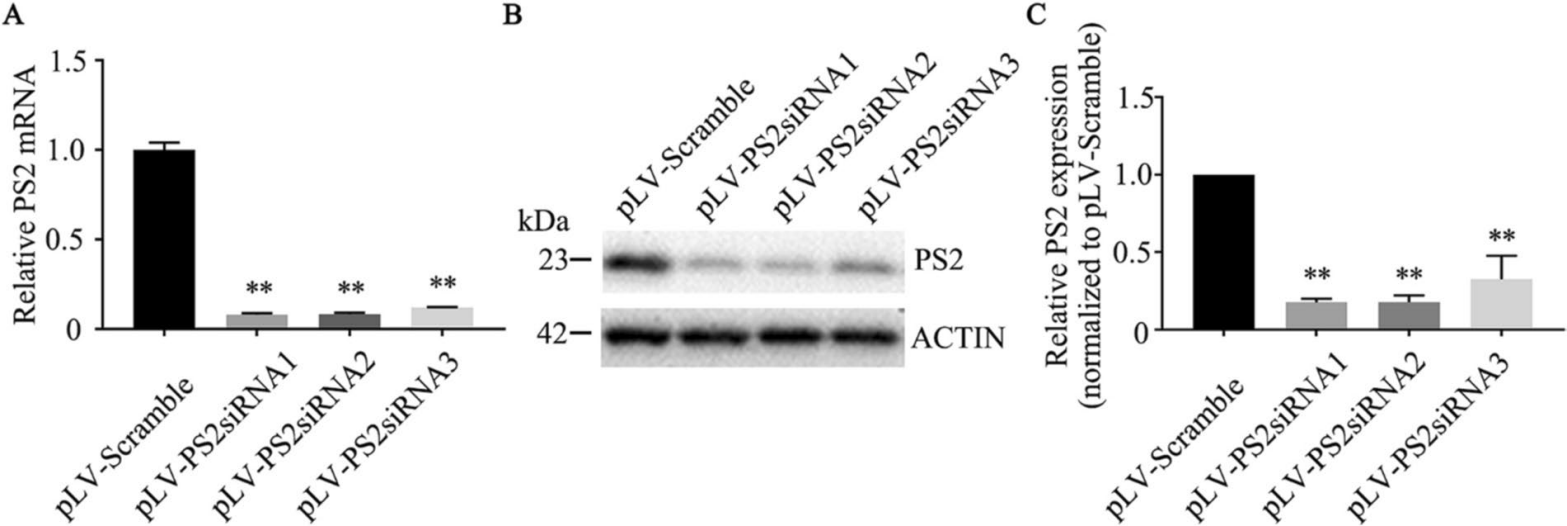
Validation of PS2 siRNA lentiviral knockdown efficiency. **A** *PS2* mRNA expression level in SH-SY5Y cells infected with pLV-PS2siRNA1, pLV-PS2siRNA2, pLV-PS2siRNA3 and pLV-scramble (*n* = 3; three independent experiments with one sample per experiment; one-way ANOVA). **B** PS2 protein expression in SH-SY5Y cells infected with pLV-PS2siRNA1, pLV-PS2siRNA2, pLV-PS2siRNA3 and pLV-scramble. **C** Bar graph showing relative PS2 expression (fold change). (*n* = 3; three independent experiments with one sample per experiment; one-way ANOVA). The data are presented as the mean ± standard deviation (mean ± SD) values; *P < 0.05, **P < 0.01

Three siRNAs to individually knock down *PS2* mRNA levels in SH-SY5Y cells were examined. Using qRT‒PCR analysis, we found that pLV-PS2-siRNA1 knocked down *PS2* mRNA expression most significantly (the interference efficiencies of the pLV-PS2-siRNA1, pLV-PS2-siRNA2, and pLV-PS2-siRNA3 lentiviral vectors were 92.16%, 91.86%, and 88.18%, respectively, compared with the pLV-scramble vector (Fig. 1A). Therefore, pLV-PS2-siRNA1 was used for the subsequent experiments. In addition, the endogenous PS2 level in SH-SY5Y cells infected with pLV-PS2-siRNA lentivirus was measured, and the results showed that pLV-PS2-siRNA1 lentivirus had the best interference effect (Fig. 1B, C).

### Analysis of Transcriptome Sequencing Results

**Fig. 2:**
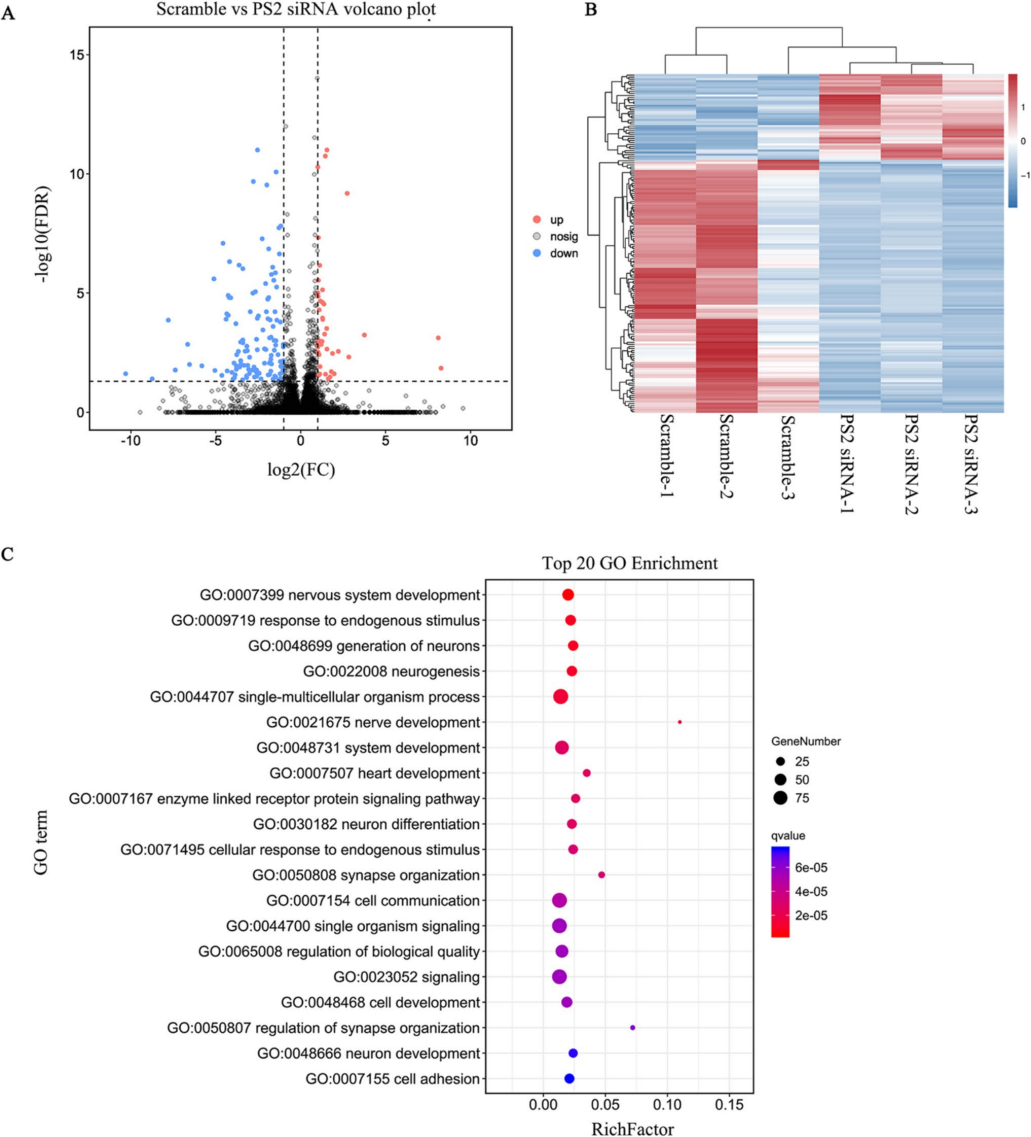
Volcano plot and heatmap showing differential expression in the scramble and PS2 siRNA groups.** A** Volcano plot of PS2 siRNA samples against scramble samples. **B** Heatmap showing DEGs between the scramble and PS2 siRNA groups. Each column represents a sample, and each row represents a gene. The colors represent the expression levels of genes in each sample. **C** Bubble diagram of the top 20 enriched GO biological processes. The bubble size is proportional to the number of genes. The redder the color, the smaller is the *Q* value

To explore the mechanism of PS2 in the pathogenesis of AD, we used RNA-seq analysis based on high-throughput sequencing to investigate the transcript changes in PS2 siRNA-transfected and scramble siRNA-transfected SH-SY5Y cells. A total of 166 DEGs were identified, among which 42 were upregulated and 124 were downregulated (Fig. 2A). The cluster patterns of the PS2 siRNA and scramble groups were significantly different (Fig. 2B). To further clarify the basic functions of the related target mRNAs based on the functional classification of DEGs, GO functional enrichment analysis was performed. The biological processes (BPs) were associated with the following terms: single organization process, cellular process, biological regulation, positive regulation of biological process and response to stimulus, etc. The cellular components (CCs) were associated with the following terms: cell, cell part, cell membrane, organelle, membrane part, synapse, synapse part, etc. The molecular functions (MFs) were associated with the following terms: cell binding, cell catalytic activity, molecular transducer activity, molecular function regulator and protein binding, etc. (Supplementary Fig. 1).

Then, we performed an analysis on the top 20 GO terms in the biological process category, and the results showed that nervous system development was the GO term most significantly enriched in the upregulated genes. The terms generation of neurons, neurogenesis, nerve development, neuron differentiation, synapse organization, regulation of synapse organization, neuron development, and terms related to other biological processes of the nervous system were the main terms enriched in the other DEGs (Fig. 2C).

The main biochemical metabolic pathways and signal transduction pathways in which DEGs are involved can be determined through identification of significant enrichment of KEGG pathways. KEGG pathway enrichment analysis showed that 72 candidate pathways were enriched in the DEGs between the scramble and PS2 siRNA groups. Among the top 20 enriched pathways, the serotonergic synapse pathway was the most significantly enriched. The hippocampal signaling pathway, dopaminergic synapse, synaptic vesicle cycle, gamma-aminobutyric acid (GABA)ergic synapse, and Rap1 signaling pathway were also significantly enriched (Supplementary Fig. 2).

Further analysis of these DEGs showed that multiple KEGG pathways were enriched in a class of genes that regulate GTPase binding or GTPase activity, containing genes such as *G protein subunit Gamma 3* (*GNG3*), *Jun proto oncogene/AP-1 transcription factor subunit* (*JUN*), *Reelin* (*RELN*), and *Rap guanine nucleotide exchange factor 5* (*RAPGEF5*) (see Table 2), suggesting that these DEGs related to the regulation of GTPase activity may play important biological roles.

**Table 2:** Pathway notes

Scramble vs PS2-siRNA (*n*)	All (*n*)	Pathway	Genes	*P* value	*Q* value
5	115	Serotonergic synapse	GNG3; GABRB2; KCND2; SLC18A1; MAOB	0.003103395	0.3956533
4	91	Complement and coagulation cascades	CD55; VWF; C7; PLAT	0.007835499	0.3956533
3	49	Cocaine addiction	SLC18A1; MAOB; JUN	0.00862324	0.3956533
4	95	Morphine addiction	GNG3; PDE3A; GABRB2; GABRG2	0.009095479	0.3956533
5	178	Tight junction	PARD6B; RUNX1; PPP2R2B; MSN; JUN	0.01873816	0.4592755
3	70	Drug metabolism cytochrome P450	MGST1; UGT2B4; MAOB	0.02252871	0.4592755
3	70	Amphetamine addiction	SLC18A1; MAOB; JUN	0.02252871	0.4592755
2	29	Hippo signaling pathway — multiple species	FAT4; DCHS2	0.02581512	0.4592755
4	135	Purine metabolism	GUCY1A2; PDE3A; ENPP4; ENPP1	0.02924684	0.4592755
4	136	Dopaminergic synapse	GNG3; PPP2R2B; SLC18A1; MAOB	0.0299413	0.4592755
3	79	Synaptic vesicle cycle	SLC1A2; SLC6A2; SLC18A1	0.03080993	0.4592755
4	140	Apelin signaling pathway	RYR2; APLNR; GNG3; PLAT	0.03281695	0.4592755
4	142	Fluid shear stress and atherosclerosis	MGST1; VEGFA; PLAT; JUN	0.03431369	0.4592755
8	447	PI3K-Akt signaling pathway	DDIT4; GNG3; VWF; ERBB4; PPP2R2B; VEGFA; RELN; LPAR1	0.03848891	0.4599038
2	37	Starch and sucrose metabolism	ENPP1; GYG2	0.04051757	0.4599038
3	91	GABAergic synapse	GNG3; GABRB2; GABRG2	0.04400953	0.4599038
5	226	Rap1 signaling pathway	ID1; PARD6B; VEGFA; RAPGEF5; LPAR1	0.04565179	0.4599038
2	41	Nicotine addiction	GABRB2; GABRG2	0.04880135	0.4599038
3	96	TGF-beta signaling pathway	ID1; INHBA; ID3	0.05021938	0.4599038
4	167	Wnt signaling pathway	DKK1; LGR5; SFRP1; JUN	0.05636153	0.4709099

**Fig. 3:**
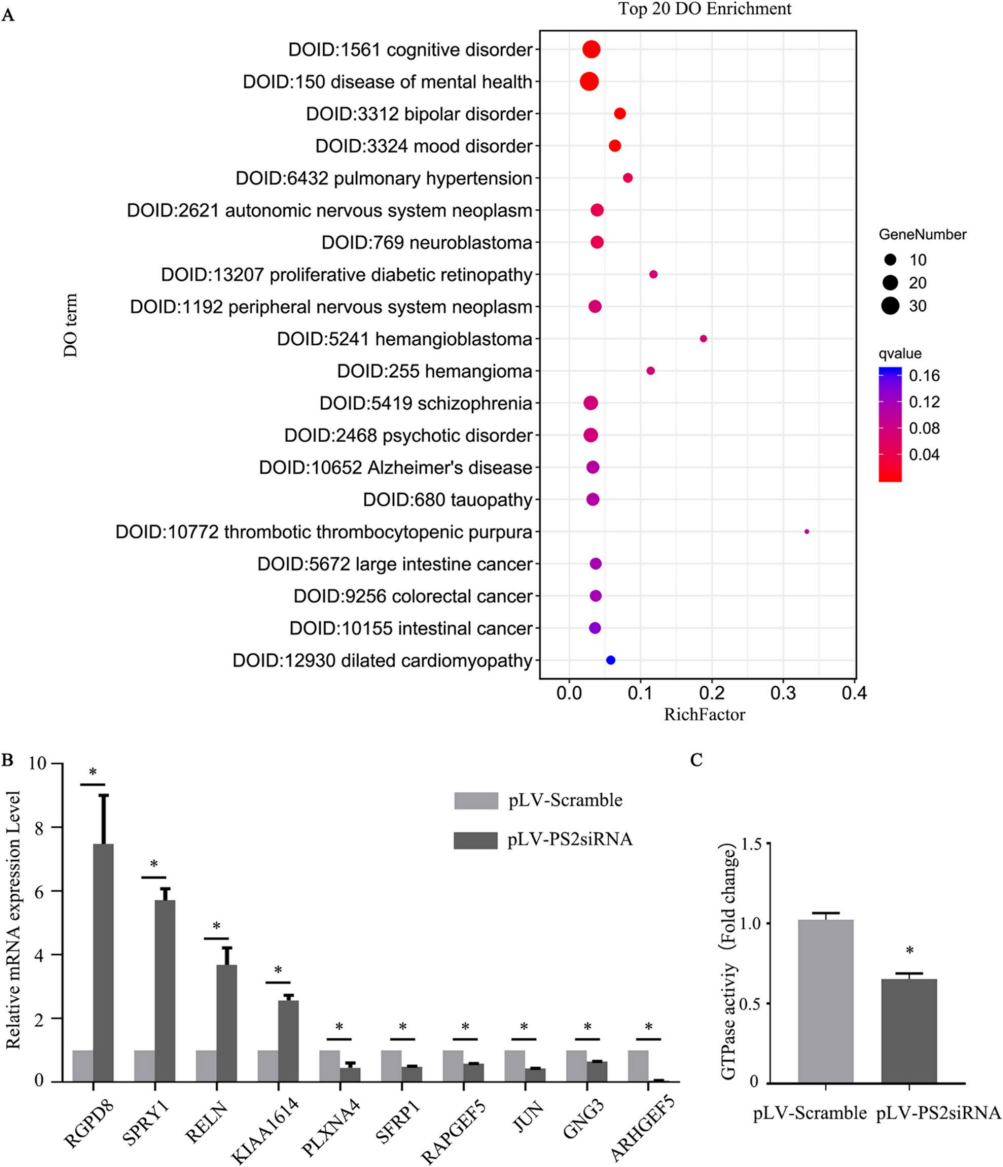
Bubble diagram of DO enrichment analysis results, verification of RNA sequencing results by qRT‒PCR, and GTPase activity measurement in SH-SY5Y cells. **A** Bubble diagram of the top 20 enriched DO terms.** B** qRT‒PCR was performed to verify the DEGs related to GTPase regulation in PS2 siRNA samples compared to scramble samples (*n* = 3; three independent experiments with one sample per experiment; one-way ANOVA). The data are presented as the means ± SDs; *P < 0.05. **C** Compared with that in pLV-scramble-transfected cells, the GTPase activity in PS2 siRNA-transfected SH-SY5Y cells was significantly decreased (*n* = 3; three independent experiments with one sample per experiment; one-way ANOVA). The data are presented as the means ± SDs; *P < 0.05

In addition, all DEGs were mapped to DO terms in the DO database. The most significantly enriched DO item was cognitive disorder. In addition, these DEGs were also involved in the occurrence and development of AD (Fig. 3A).

### mRNA Expression and Significance of GTPase-Related DEGs

According to the screening and analysis of the DEGs in the PS2 siRNA cell transcriptome data, PS2 may be related to the regulation of GTPases (see Table 3). Multiple KEGG pathways, such as serotonergic synapse and Rap1 signaling pathway, were enriched in genes related to GTPase regulation, such as *GNG3*, *JUN*, *RELN*, and *RAPGEF5*.

**Table 3:** Statistical table of DEGs related to GTPase regulation

Symbol	log2(fc)	FDR	GO function/process
RGPD8	1.726020088	< 0.05	GO:0005096//GTPase activator activity; GO:0043547//positive regulation of GTPase activity;
SPRY1	1.625270489	< 0.05	GO:0034260//negative regulation of GTPase activity;
RELN	1.110806786	< 0.05	GO:0034260//negative regulation of GTPase activity;
KIAA1614	1.073794074	< 0.05	GO:0017048//Rho GTPase binding
PLXNA4	− 1.19710442	< 0.05	GO:0043087//regulation of GTPase activity;
SFRP1	− 1.25907173	< 0.05	GO:0043547//positive regulation of GTPase activity;
RAPGEF5	− 1.4265983	< 0.05	GO:0007264//small GTPase mediated signal transduction;GO:0030742//GTP-dependent protein binding
JUN	− 1.49471792	< 0.05	GO:0005096//GTPase activator activity;GO:0043547//positive regulation of GTPase activity;
GNG3	− 1.91177282	< 0.05	GO:0003924//GTPase activity
ARHGEF5	− 3.12553088	< 0.05	GO:0090630//activation of GTPase activity; GO:0043087//regulation of GTPase activity;GO:0043547//positive regulation of GTPase activity;GO:0051056//regulation of small GTPase mediated signal transduction;GO:0005089//Rho guanyl-nucleotide exchange factor activity;GO:0005525//GTP binding;

The disease term most strongly enriched in the DEGs in DO enrichment analysis was cognitive dysfunction (Fig. 3A), which is also related to the pathogenesis of AD, suggesting that when PS2 gene expression was knocked down, these DEGs related to the regulation of GTPases may play an important biological role, which may eventually lead to cognitive dysfunction or the occurrence of AD.

We next confirmed the expression of the genes regulating GTPases by qPCR, and the verification results were consistent with the RNA-seq results (Fig. 3B). In PS2 siRNA-transfected SH-SY5Y cells, the mRNA levels of the positive regulators of GTPase activity *Ran binding protein 2-like and glutamate receptor interaction domain 8* (*RGPD8*) and the negative regulators of GTPase activity *sprout receptor tyrosine kinase signal antagonist 1* (*SPRY1*) and *RELN* were significantly increased (*P* < 0.05). However, the expression of *ARHGEF5*, *JUN*, *secretory frizzled–related protein 1* (*SFRP1*) and other genes that positively regulate GTPase activity was decreased significantly (*P* < 0.05) (Fig. 3B). Moreover, GTPase activity in PS2 siRNA-transfected SH-SY5Y cells was significantly lower than that in scramble siRNA-transfected cells (*P* < 0.05) (Fig. 3C).

### Expression of GTPase-Related Genes and GTPase Activity in PS2 D439A Mutant Cells

**Fig. 4:**
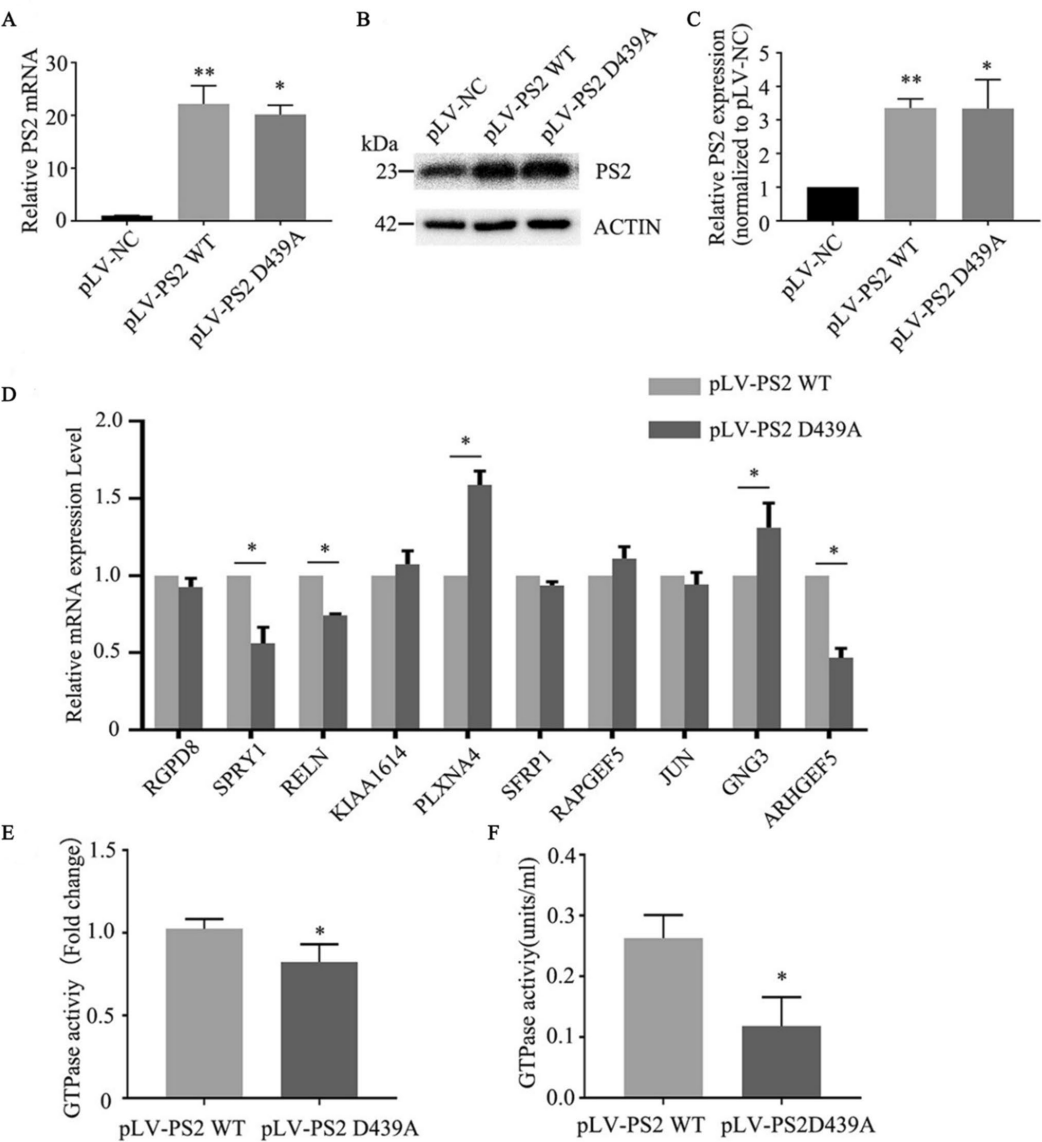
Expression of GTPase-related genes and level of GTPase activity in PS2 D439A mutant cells.** A** qPCR analysis of PS2 mRNA expression in stably transduced SH-SY5Y cell lines expressing PS2 WT or the PS2 D439A mutant (*n* = 3; three independent experiments with one sample per experiment; one-way ANOVA).** B**, **C** The protein levels of PS2 in stably transduced SH-SY5Y cell lines expressing PS2 WT, PS2 D439A mutant and empty vector control were measured by Western blotting (*n* = 3; three independent experiments with one sample per experiment; one-way ANOVA). **D** Verification of the DEGs related to GTPase regulation identified by RNA-seq in PS2 siRNA-transfected cells by qRT‒PCR in PS2 WT and PS2 D439A mutant cells (*n* = 3; three independent experiments with one sample per experiment; one-way ANOVA). **E** The GTPase activity in pLV-PS2 D439A mutant SH-SY5Y cells was compared with that in pLV-PS2 WT cells. GTPase activity was measured at room temperature, the reaction time was 30 min, and the absorbance was measured at 590 nm (*n* = 3; three independent experiments with one sample per experiment; one-way ANOVA). **F** Comparison of Miro2 GTPase activity in pLV-PS2 D439A mutant SH-SY5Y cells with that in the pLV-PS2 WT group. GTPase activity was measured at room temperature for 30 min. Finally, the absorbance was measured at 650 nm. The catalytic GTPase activity of Miro2 was calculated based on the generated standard curve (*n* = 3; three independent experiments with one sample per experiment; one-way ANOVA). The data are presented as the means ± SDs; compared with pLV-PS2 WT, *P < 0.05, **P < 0.01

We successfully established stably transduced SH-SY5Y cell lines expressing PS2 WT, PS2 D439A, or empty vector. The results of qPCR and Western blot analyses showed that *PS2* mRNA (Fig. 4A) and the PS2 protein (Fig. 4B, C) were significantly overexpressed in the stable pLV-PS2 WT-transduced and pLV-PS2 D439A-transduced cell lines compared with the empty vector-transduced cell line. The DEGs related to the regulation of GTPases identified by RNA-seq in PS2 siRNA-transfected SH-SY5Y cells were verified in PS2 D439A mutant SH-SY5Y cells. Compared with those in PS2 WT cells, the mRNA level of *ARHGEF5*, which is related to the positive regulation of GTPase activity, was significantly reduced by more than twofold, and the mRNA levels of *SPRY1* and *RELN*, which are related to the negative regulation of GTPase activity, were also reduced (*P* < 0.05). However, the mRNA expression of plexin A4 (*PLXNA4*) and *GNG3*, which are related to the regulation of GTPase activity, was increased significantly (*P* < 0.05) (Fig. 4D). We evaluated the changes in GTPase activity in these cells and found that the GTPase activity in PS2 D439A mutant SH-SY5Y cells was significantly lower than that in PS2 WT SH-SY5Y cells (*P* < 0.05) (Fig. 4E).

Recent studies have found that Miro1/2 plays a central role in mitochondrial fusion/fission and mitochondrial morphological regulation through interactions with Mfn1/2 and Drp1[11, 21], which are affected by GTPase activity [9]. Therefore, maintaining the balance of mitochondrial fusion/fission requires an appropriate level of Miro2 GTPase activity. In our study, we found that the Miro2 GTPase activity in PS2 D439A mutant cells was significantly lower than that in PS2 WT cells (*P* < 0.05) (Fig. 4F).

### Binding Analysis Between PS2 and Miro1/2

**Fig. 5:**
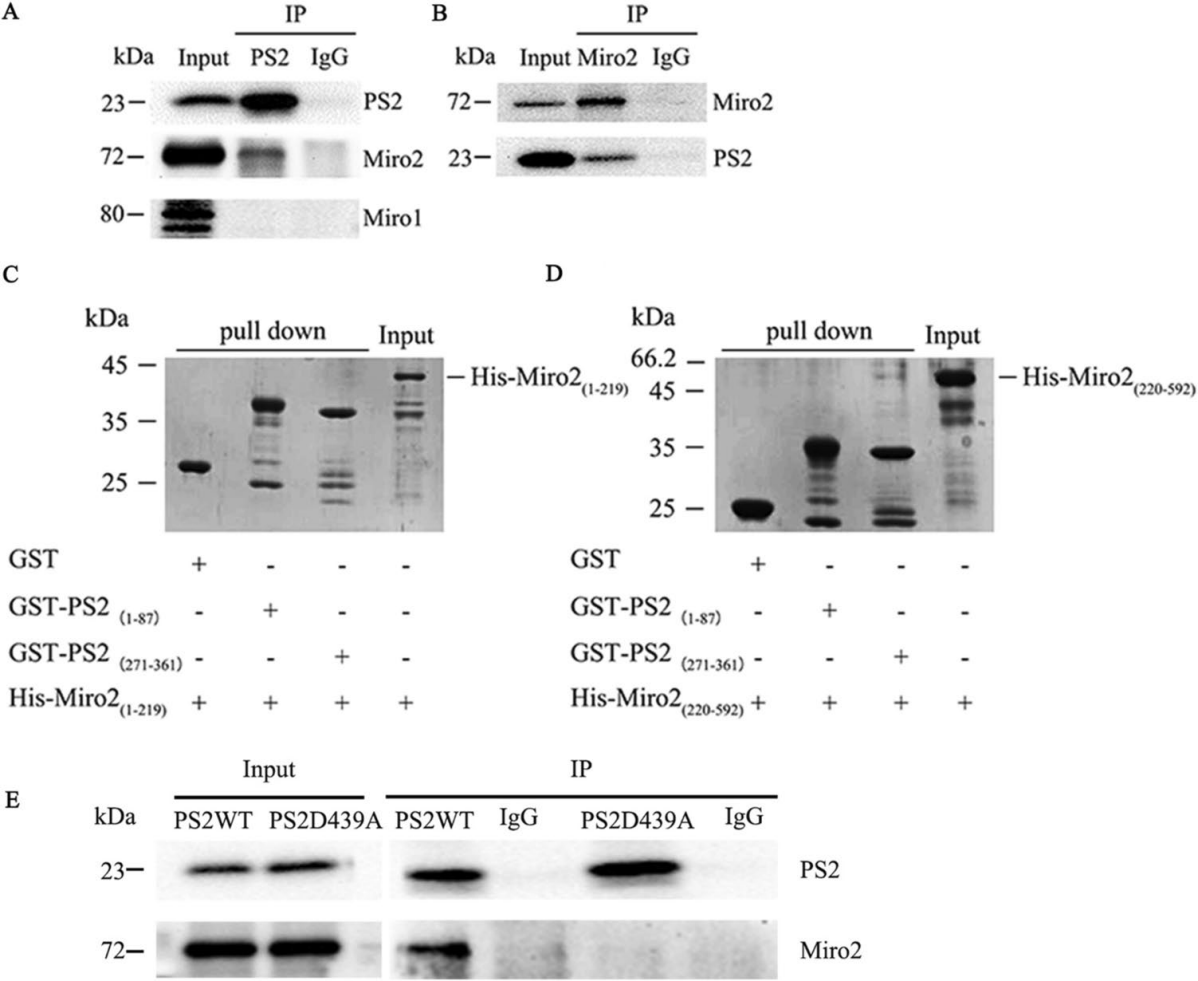
The binding ability between PS2 and Miro1/2.** A**, **B** Detergent lysates from SH-SY5Y cells expressing PS2 were immunoprecipitated (IP) with anti-Miro1, anti-Miro 2, or anti-PS2 antibodies or control IgG. PS2 coimmunoprecipitated with Miro2 but not Miro1. Miro2 also coimmunoprecipitated with PS2, as detected by Western blotting using anti-PS2 and anti-Miro2 antibodies. **C**, **D** SDS‒PAGE images of the immunoprecipitates indicating the protein domains involved in the interaction between PS2 and Miro2.** E** Detergent lysates from SH-SY5Y cells expressing PS2 WT and PS2 D439A were immunoprecipitated (IP) with an anti-Miro2 antibody or control IgG. PS2 coimmunoprecipitated with Miro2, as detected by Western blotting using anti-PS2 and anti-Miro2 antibodies, in PS2 WT cells, but in PS2 D439A mutant cells, this interaction was significantly attenuated

To verify whether PS2 can interact with Miro1/2, we first used a Co-IP experiment to detect this binding interaction. The results indicated that endogenous PS2 can interact with Miro2 but not Miro1 (Fig. 5A). In addition, Miro2 coimmunoprecipitated with PS2 (Fig. 5B). Then, we used a pulldown assay to determine whether there is a direct binding interaction between PS2 and Miro2 and to identify the interacting regions. PS2 is composed of 448 amino acids and contains 7 to 9 transmembrane domains in different organelles [3, 22]. Based on the characteristics of the transmembrane structure and the possible interaction between the extracellular domain of PS2 and Miro2, GST fusion proteins containing each of the two domains of PS2 (amino acids 1–87 and 271–361) were constructed (GST-PS2_(1–87)_ and GST-PS2_(271–361)_, respectively). Miro2 is composed of 618 amino acids and has a single transmembrane domain, and a full-length Miro2 protein could not be constructed. Thus, His fusion proteins containing each of the two domains of Miro2 (amino acids 1–219 and 220–592) were constructed (His-Miro2_(1–219)_ and His-Miro2_(220–592)_, respectively). These two domains of Miro2 contain two GTPase domains. The results of pulldown experiments suggested that GST-PS2_(271–361)_ did bind to His-Miro2_(220–592)_ but not to His-Miro2_(1–219)_ and that GST-PS2_(1–87)_ did not interact with either His-Miro2_(1–219)_ or His-Miro2_(220–592)_ (Fig. 5C, D). The above results suggested that PS2 can interact with Miro2.

To explore the potential pathological mechanism underlying the effect of the PS2 D439A mutation on mitochondrial dynamic abnormalities in the pathogenesis of AD, we next examined whether the PS2 D439A mutation could weaken the interaction between PS2 and Miro2 and then dysregulate mitochondrial dynamics. First, we found that overexpression of PS2 D439A significantly attenuated the interaction between PS2 and Miro2 (Fig. 5E).

### PS2 D439A Mutation Adversely Affected Mitochondrial Morphology and the Dysfunction of Mitochondrial Fusion and Fission Dynamics

**Fig. 6:**
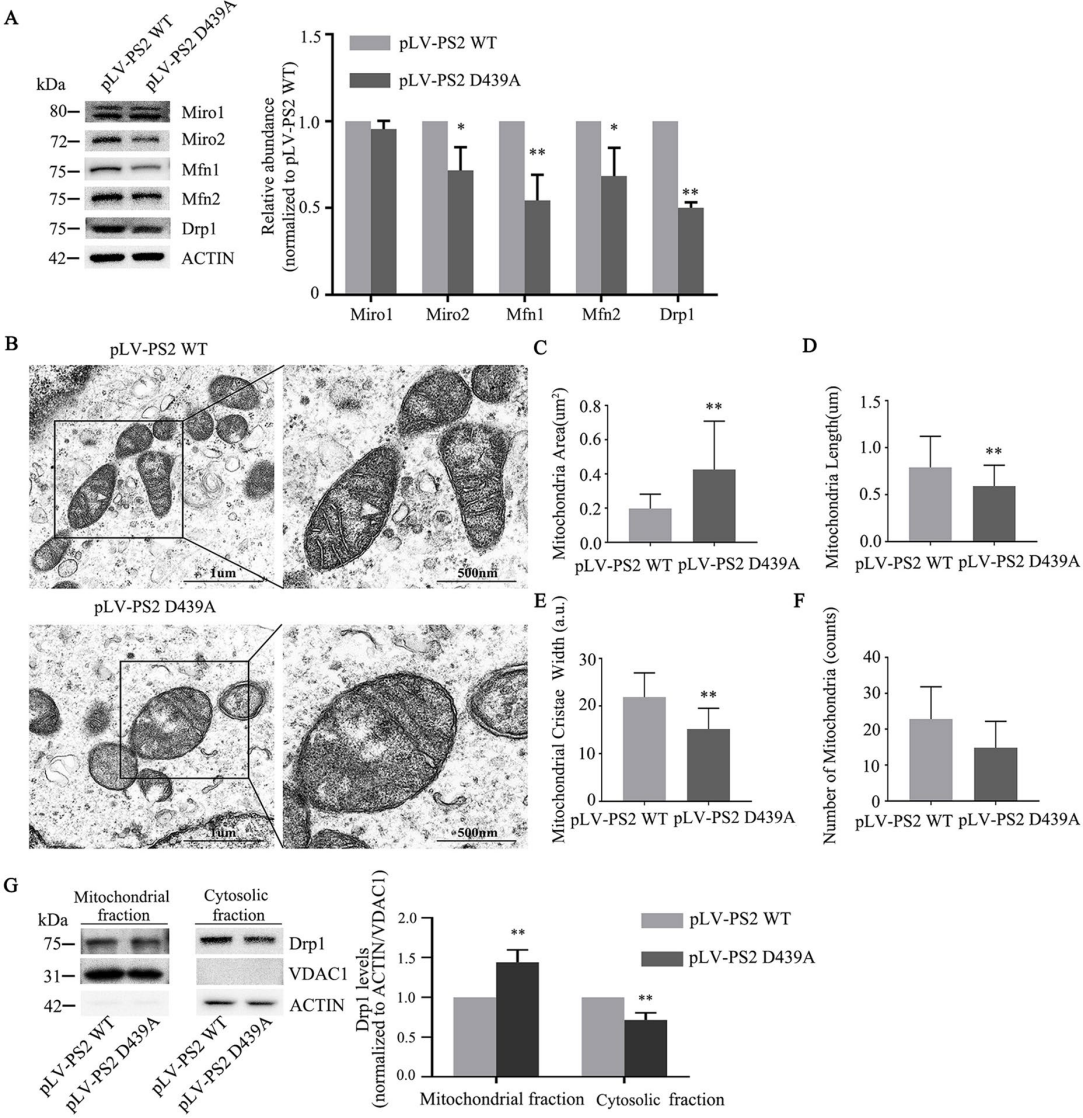
The PS2 D439A mutation affects the balance of mitochondrial fusion and fission and mitochondrial morphology. **A** The expression of Miro1, Miro2, Mfn1, Mfn2 and Drp1 was determined by quantitative Western blot analysis (*n* = 3; three independent experiments with one sample per experiment; one-way ANOVA). **B** TEM of mitochondria in SH-SY5Y cells transduced with pLV-PS2 WT and pLV-PS2 D439A mutant (*n* = 3; three independent experiments with one sample per experiment; one-way ANOVA). **C** Bar graph showing the average mitochondrial surface area determined from 22 mitochondria in SH-SY5Y cells transduced with pLV-PS2 WT and from 21 mitochondria in pLV-PS2 D439A mutant-transduced cells. **D** Bar graph showing the average mitochondrial length determined from 33 mitochondria in SH-SY5Y cells transduced with pLV-PS2 WT and from 35 mitochondria in pLV-PS2 D439A-transduced cells. **E** Bar graph showing the average mitochondrial cristae width determined from 22 mitochondria in SH-SY5Y cells transduced with pLV-PS2 WT and from 21 mitochondria in pLV-PS2 D439A-transduced cells.** F** Bar graph showing the average number of mitochondria in SH-SY5Y cells transduced with pLV-PS2 WT and pLV-PS2 D439A. Scale bars, 1 μm in the left panels and 500 nm in the right panels. **G** Western blot analysis of Drp1 expression in the mitochondrial and cytosolic fractions in SH-SY5Y cells overexpressing PS2 WT and the PS2 D439A mutant (*n* = 3; three independent experiments with one sample per experiment; one-way ANOVA). The data are presented as the means ± SDs; compared with pLV-PS2 WT, *P < 0.05, **P < 0.01

We next examined whether the PS2 D439A mutant can affect the expression of Miro1/2. The PS2 D439A mutation decreased the expression of Miro2 but not Miro1, and we also observed that the expression of Mfn1, Mfn2 and Drp1 decreased significantly (Fig. 6A). Next, by TEM, we observed that PS2 D439A mutant overexpression caused mitochondrial morphology changes (Fig. 6B), highlighted by the significantly increased mean mitochondrial area (0.43 ± 0.06 µm^2^ vs. 0.20 ± 0.02 µm^2^) (Fig. 6C). Several swollen mitochondria were observed, whereas the mean width of mitochondrial cristae (15.19 ± 0.95 a.u. vs. 21.88 ± 1.08 a.u.) (Fig. 6E) and mean length of mitochondria (0.59 ± 0.04 µm vs. 0.79 ± 0.06 µm) (Fig. 6D) were significantly decreased compared with those in cells expressing PS2 WT. The mean number of mitochondria per cell in the PS2 D439A mutant group (14.80 ± 3.30) was less than that in the PS2 WT group (22.80 ± 4.03), but the difference was not statistically significant (Fig. 6F). The overall state of the cells and nucleus  was also detected by TEM (Supplementary Fig. 3).

The TEM results showed that the decrease in the average length of mitochondria in PS2 D439A mutant cells indicated an increase in mitochondrial fission, while Western blotting showed that the expression of total Drp1 protein was decreased. Because the function of Drp1 in dividing mitochondria is initiated mainly after its translocation from the cytoplasm to the OMM, the amount of Drp1 causing mitochondrial fission is determined by the number of Drp1 proteins in the OMM but not by the number of Drp1 proteins in the cytoplasm [23]. Therefore, we isolated, extracted, and purified total mitochondrial protein and compared the differences in Drp1 expression between mitochondria and the cytoplasm. The expression of Drp1 in mitochondria was higher in PS2 D439A mutant cells than in PS2 WT cells; thus, mitochondrial fission in PS2 D439A mutant cells may be increased (Fig. 6G).

### Role of PS2 and Miro2 in Mitochondrial Fusion and Fission

**Fig. 7:**
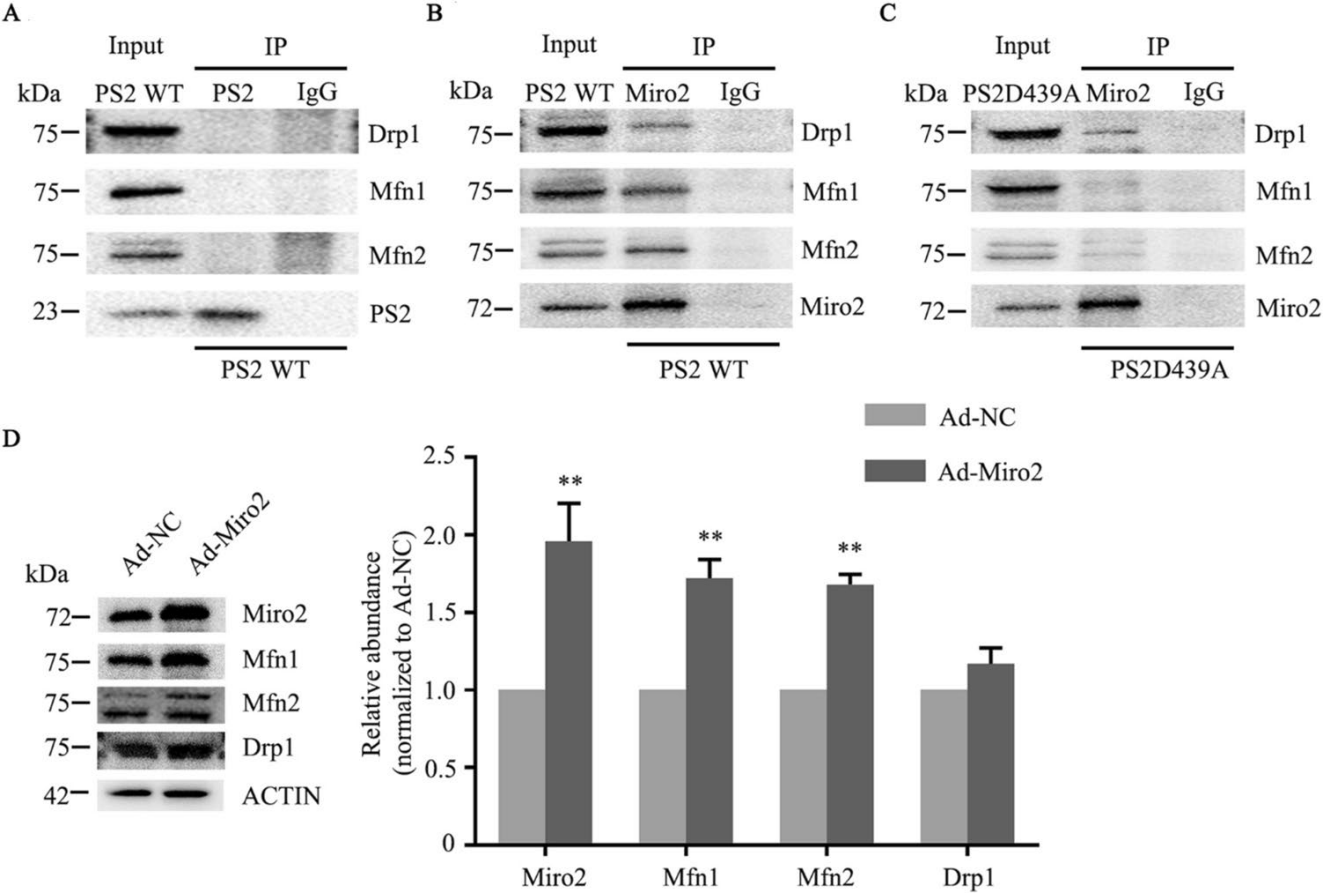
Role of PS2 and Miro2 in mitochondrial fusion and fission.** A** Protein lysates of SH-SY5Y cells with stable expression of PS2 WT, Mfn1, Mfn2, and Drp1 were immunoprecipitated with an anti-PS2 antibody, with normal IgG as a control. PS2 could not bind to Mfn1, Mfn2, or Drp1.** B** Protein lysates of SH-SY5Y cells with stable expression of PS2 WT, Mfn1, Mfn2 and Drp1 were immunoprecipitated with an anti-Miro2 antibody, and normal IgG was used as the control. Miro2 bound to Mfn1, Mfn2, and Drp1. **C** Protein lysates of SH-SY5Y cells with stable expression of PS2 D439A, Mfn1, Mfn2 and Drp1 were immunoprecipitated with an anti-Miro2 antibody, with normal IgG as a control. Overexpression of the PS2D439A mutant decreased Miro2/Mfn1 and Miro2/Mfn2 protein binding but not Miro2/Drp1 binding.** D** PS2 D439A mutant SH-SY5Y cells were infected with Ad-Miro2 and Ad-NC, and protein was extracted 48 h after infection. Compared with Ad-NC, Ad-Miro2 significantly increased the expression of Miro2, Mfn1 and Mfn2, but there was no significant difference in the expression of Drp1. The gray values of the bands were analyzed semiquantitatively, and the protein levels in the cells infected with Ad-NC were standardized to 1.0 (*n* = 3; three independent experiments with one sample per experiment; one-way ANOVA). The data are presented as the means ± SDs; compared with the Ad-NC group, *P < 0.05, **P < 0.01

We explored whether PS2 can interact with Mfn1, Mfn2, and Drp1 by using Co-IP. The results showed that PS2 could not interact with Mfn1, Mfn2, or Drp1 (Fig. 7A), while Miro2 could interact with Mfn1, Mfn2, and Drp1 (Fig. 7B). Previous studies found that the changes in Mfn1 and Mfn2 expressions may be regulated by Miro2, which promotes the fusion of mitochondria through its interactions with Mfn1/2 [11, 24]. We performed another Co-IP assay to explore the changes in the binding between Miro2 and the above mitochondrial dynamics-related proteins after introduction of the PS2 D439A mutation. The binding degrees of Miro2 to Mfn1/Mfn2 were decreased in cells overexpressing the PS2 D439A mutant, while the binding degree of Miro2 to Drp1 did not change significantly in these cells (Fig. 7C).

To further explore whether the changes in Mfn1/2 and Drp1 expression in PS2 D439A mutant cells were caused by changes in Miro2 expression, we infected PS2 D439A mutant SH-SY5Y cells with Ad-Miro2. The levels of Mfn1 and Mfn2 increased significantly with increasing Miro2 expression in Ad-Miro2-infected PS2 D439A mutant cells compared with Ad-NC-infected PS2 D439A mutant cells, but there was no significant difference in the change in Drp1 expression between these cells (Fig. 7D).

### PS2 D439A Mutation Affects Mitochondrial Function

**Fig. 8:**
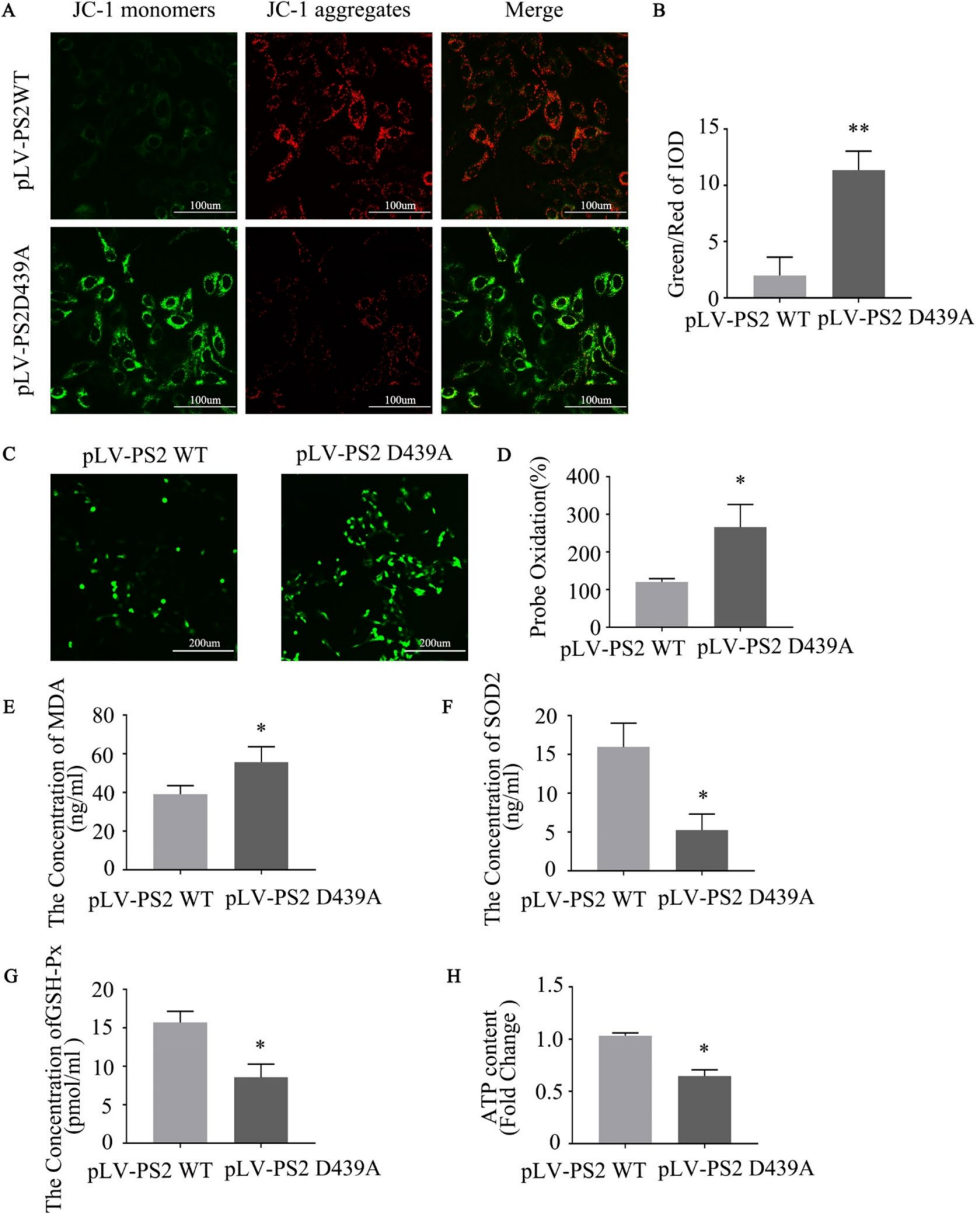
The PS2 D439A mutation affects mitochondrial function. **A** Detection of JC-1 signals in SH-SY5Y cells expressing PS2 WT and the PS2 D439A mutant by fluorescence microscopy.** B** The data are expressed as the ratio of green fluorescence/red fluorescence integrated optical density (IOD) values (*n* = 3; three independent experiments with one sample per experiment; one-way ANOVA)** C** Measurement of ROS levels in SH-SY5Y cells expressing PS2 WT and the PS2 D439A mutant. **D** The data are expressed as probe oxidation (%). **E** Up-regulation of MDA in PS2 D439A mutant cells was validated by ELISAs. **F** Down-regulation of SOD2 in PS2 D439A mutant cells was validated by ELISAs. **G** Down-regulation of GSH-Px in PS2 D439A mutant cells was validated by ELISAs. **H** Measurement of ATP levels in SH-SY5Y cells expressing PS2 WT and the PS2 D439A mutant. (*n* = 3; three independent experiments with one sample per experiment; one-way ANOVA). The data are presented as the means ± SDs, compared with the pLV-PS2 WT group, *P < 0.05, **P < 0.01

The dysfunction of mitochondrial fusion/fission dynamics in defective mitochondria can lead to ultrastructural defects, which in turn may have deleterious effects on MMP [25]. Therefore, we applied JC-1 staining to detect the changes in MMP after introduction of the PS2 D439A mutation. The MMP in PS2 D439A mutant cells was significantly lower than that in PS2 WT cells (Fig. 8A, B). These results indicated that mitochondrial functions were impaired in PS2 D439A mutant cells.

Mitochondrial dynamics are essential for maintaining mitochondrial integrity and functions, including regulation of ROS generation and apoptosis, and excessive mitochondrial fission and/or subsequent mitochondrial structural damage are associated with increased ROS production [25]. Oxidative stress is considered a primary event in the progression of AD [26]. Measurement of ROS levels after introduction of the PS2 D439A mutation showed that ROS levels were significantly increased (Fig. 8 C, D). In addition, we estimated MDA, SOD2, GSH-Px, and ATP levels in PS2 WT and PS2 D439A mutant cells. SOD2, GSH-Px, and ATP levels were decreased, while, MDA levels were increased markedly in the PS2 D439A mutant cells compared with PS2 WT cells (Fig. 8E, F, G, H).

### PS2 D439A Mutation Decreases Cell Viability and Induces Apoptosis

**Fig. 9:**
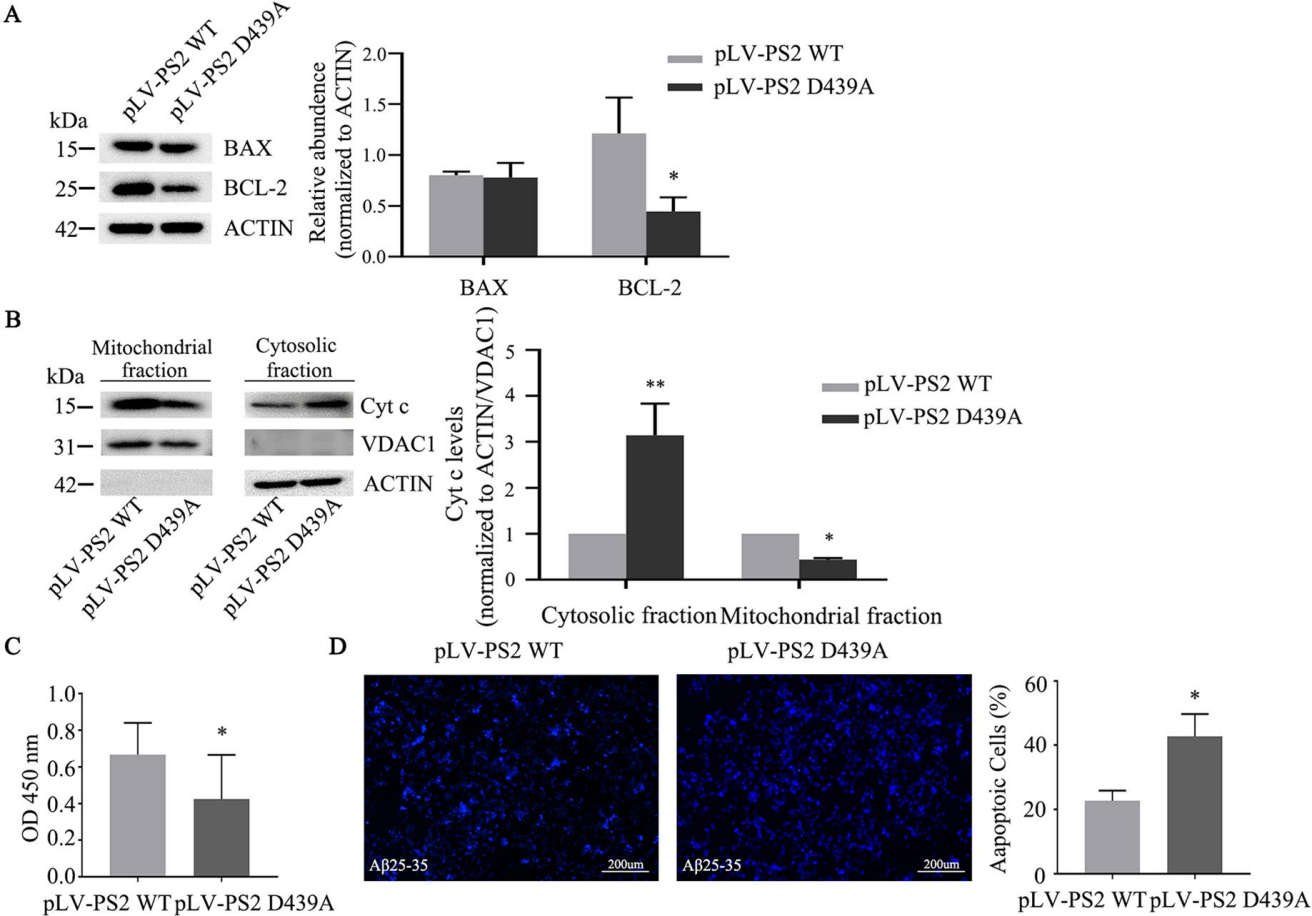
The PS2 D439A mutation affects cell viability and induces apoptosis.** A** Protein expression levels of BCL-2 and BAX in SH-SY5Y cells overexpressing PS2 WT and the PS2 D439A mutant. Quantitative analysis of data from three independent experiments was performed to assess expression levels. **B** Protein expression levels of Cyt c in the mitochondrial and cytosolic fractions in SH-SY5Y cells overexpressing PS2 WT and the PS2 D439A mutant. The ACTIN and VDAC1 proteins were used as loading controls, and quantitative analysis of data from three independent experiments was performed to assess expression levels. **C** The viability of SH-SY5Y cells expressing PS2 WT and the PS2 D439A mutant was measured by a CCK8 assay (*n* = 3; three independent experiments with one sample per experiment; one-way ANOVA). **D** Apoptotic cells were detected by staining with the nuclear dye Hoechst 33,342, which reveals fragmented or intensely stained nuclei (*n* = 3; three independent experiments with one sample per experiment; one-way ANOVA). The data are presented as the means ± SDs; compared with the pLV-PS2 WT group, *P < 0.05, **P < 0.01

Disrupted mitochondrial dynamics and increased ROS generation may induce apoptosis, and many signals for cellular apoptosis are regulated by BCL-2 family proteins and converge at mitochondria [27]. Next, we examined BCL-2 and BAX expression levels. In SH-SY5Y cells expressing the PS2 D439A mutant, the BCL-2 level was significantly decreased compared with that in PS2 WT cells, but no significant change was observed in the level of BAX. Quantification of protein band densities indicated that in PS2 D439A mutant cells, the BCL-2 expression level was less than 50% of that in PS2 WT cells (Fig. 9A). The mitochondrial apoptotic pathway regulated by the BCL-2 family of proteins promotes mitochondrial outer membrane permeabilization (MOMP), which allows the release of proapoptotic factors such as cytochrome c (Cyt c) from mitochondria into the cytosol to activate the caspase cascade [28]. Therefore, we further examined the Cyt c levels in the mitochondrial and cytosolic fractions in SH-SY5Y cells overexpressing PS2 WT and the PS2 D439A mutant. The Cyt c level was significantly increased in the cytosol and decreased in mitochondria in cells expressing the PS2 D439A mutant compared with cells overexpressing PS2 WT (Fig. 9B).

A CCK8 assay was used to evaluate cell viability after introduction of the PS2 D439A mutation and showed that the viability of SH-SY5Y cells after infection with pLV-PS2 D439A was significantly lower than that after infection with pLV-PS2 WT (Fig. 9C). A previous study found that the PS2 D439A mutation did not change the Aβ42/40 ratio or Aβ42 level [16]. To further clarify the synergistic effects of Aβ and mitochondrial dysfunction, we exposed SH-SY5Y cells infected with the pLV-PS2 WT or pLV-PS2 D439A mutant lentivirus to 40 μM Aβ25-35 for 48 h and then determined the percentage of apoptotic cells. In the AD cell model induced by Aβ25-35, apoptotic cells were detected by Hoechst 33,342 staining and observed by fluorescence microscopy. The percentage of apoptotic cells in the PS2 D439A mutant group (42.77 ± 4.02%) was significantly higher than that in the PS2 WT group (22.72 ± 1.82%) in the AD cell model induced by Aβ25-35 (*P* < 0.05) (Fig. 9D).

## Discussion

Not all pathogenic PS1/2 FAD mutations are thought to affect the Aβ42 level or Aβ42/40 ratio [17]. Some elderly people have obvious Aβ42 deposition but no cognitive impairment, indicating that the cognitive impairment in AD may be caused or modulated by factors other than insoluble forms of Aβ [29]. Therefore, we conducted transcriptome sequencing analysis on PS2 siRNA-transfected SH-SY5Y cells to explore the potential role of PS2 in the pathogenesis of AD. Subsequently, DEGs were analyzed in each KEGG pathway and a set of genes that regulate GTPase binding or GTPase activity was identified. Among them, the most significantly downregulated gene was *ARHGEF5*, which plays a pivotal role in the positive regulation of GTPase activity according to GO enrichment analysis.

Recently, diverse alterations in Rho GTPase modulation and mitochondrial functionality have been consistently reported at early stages of AD, but the underlying mechanisms are poorly understood [11, 30]. GTPase is a key signal transduction enzyme that links extracellular signals with neuronal responses required for the construction of neuronal networks, synaptic function and plasticity [31]. GTPase activity is also involved in the regulation of mitochondrial function; GTP hydrolysis is very important for the translocation of proteins into the mitochondrial matrix, and the proteins entering mitochondria regulate different pathways in mitochondria [32]. The dynamic processes of fusion and fission of mitochondria are mediated by several GTPases [33].

Miro1/2 and large GTPases such as Mfn1/2 and Drp1 have been shown to play an essential role in regulating the balance between fusion and fission to control mitochondrial morphology, and they also mediate mitochondrial homeostasis and anterograde and retrograde mitochondrial transport [11, 34]. These proteins contain GTPase domains, and their functions can be affected by GTPase activity [34]. Small GTPases, such as Ras homolog (Rho) family proteins, constitute major branches of the Ras superfamily; among them, RhoA, Rac1, and Cdc42 have been extensively studied, and they can regulate the growth of axons and are closely related to neuronal development and the molecular neuropathogenesis of AD [35]. Rho GTPases are related to almost all basic cellular processes of brain development, from neurogenesis to axon guidance [31]. Rho GTPases are also directly involved in the regulation of cell morphology, adhesion and migration [31]. The above biological processes involving regulation by Rho GTPases were highly consistent with the top 20 GO biological process terms (Fig. 2; Supplementary Fig. 1). DO enrichment analysis suggested that the changes in the expression of the DEGs eventually lead to the occurrence and development of cognitive dysfunction and AD (Fig. 3A), further suggesting that *PS2* may participate in the pathological processes related to cognitive dysfunction and AD by regulating the expression of GTPase-related genes.

Rho GTPase activity is regulated by three proteins: guanine nucleotide exchange factors (GEFs), GTPase-activating proteins (GAPs) and GDP dissociation inhibitors (GDIs) [36]. Ras superfamily members and other GTPases contain a core guanine binding domain, which can bind with high affinity to GTP and GDP and has the ability to hydrolyze GTP [36]. The activation of Rho GTPases is regulated by GEFs to exchange nonactivated GDP for activated GTP [37]. In addition, the GTP/GDP cycle can be regulated by interactions between GTPases and other proteins, and the related binding proteins activate downstream targets by increasing the influence of GEFs or inhibiting the role of GAPs[38]. Our study found that after *PS2* gene knockdown, *ARHGEF5* expression decreased significantly. *ARHGEF5* plays an important role in the regulation of endogenous Rho GTPases [38], and aberrant regulation of Rho GTPases plays a key role in neurodegenerative diseases such as AD [39]. Here, we found that GTPase activity was significantly decreased in PS2 siRNA-transfected SH-SY5Y cells.

Extensive contradictory studies have indicated that mitochondrial dysfunction exists independently of Aβ and potentially lies upstream of Aβ deposition; the authors of these studies proposed a primary mitochondrial cascade hypothesis that assumes that mitochondrial pathology hierarchically supersedes Aβ pathology [6]. Indeed, mitochondrial dysfunction was proven to be one of the earliest and most prominent features of AD [40]. The dysfunction of mitochondrial dynamics and the aberrant regulation of GTPase activity all contribute to AD pathogenesis [5, 39]. Based on the primary mitochondrial cascade hypothesis, it is very important to further explore the relationship between PS2 and  the mitochondrial dynamics-related proteins Miro1/2, Mfn1/2, and Drp1. Whether the PS2 D439A mutation affects mitochondrial fusion/fission by regulating GTPase activity deserves further study.

Then, we verified the expression of genes related to the regulation of GTPase activity in PS2 D439A mutant cells. The expression of *ARHGEF5* in PS2 D439A mutant cells was lower than that in PS2 WT cells, and the overall GTPase activity in the mutant cells was also significantly decreased. Previous studies found that large GTPases such as Mfn1/2 and Drp1 can hydrolyze GTP and regulate the GTP/GDP cycle independently of separate GEFs or GAPs [41]. However, members of the small GTPase family usually need assistance from GEFs or GAPs in releasing tightly bound GDP or enhancing GTPase activity [42]. *Vimar*, encoding an atypical GEF, can function as a GEF of Miro, in which Miro can mediate the increase in mitochondrial fission in the context of *vimar* deletion [42]. In addition to the significant decrease in the expression of *ARHGEF5*, Miro2 GTPase activity was significantly reduced in PS2 D439A mutant cells. The Rho GTPase activity of Miro is closely related to the regulation of mitochondrial fusion/fission and other physiological functions [43]. Therefore, the PS2 D439A mutation may lead to a decrease in Miro2 GTPase activity by regulating the decrease in *ARHGEF5* expression, which may then affect the balance of mitochondrial dynamics.

To verify this hypothesis, we next explored the potential functional relationship between PS2 and Miro1/2 and found that PS2 can interact with Miro2 but not Miro1. The results of pulldown experiments indicated that GST-PS2_(271–361)_ can interact specifically with His-Miro2_(220–592)_, a region containing a GTPase domain. Collectively, our data demonstrate that PS2 interacts directly with Miro2 in vitro and in cultured cells. Miro presumably interacts with Mfn1/2 or other unknown factors to influence mitochondrial morphology [21]. Other research found that Miro2 can promote mitochondrial fusion and increase the mean mitochondrial circularity and area, effects controlled by its GTPase domains [43]. The function of Miro in regulating mitochondrial morphology is conserved in plants [43] and has also been observed in Drosophila [44]. The molecular mechanism of Miro regulates mitochondrial morphology is unclear, overexpression of Miro can lead to mitochondrial aggregation excessively or fusion phenotype [21]. In this study, Co-IP experiments indicated that the PS2 D439A mutation decreased the interaction between PS2 and Miro2. Moreover, we found that the PS2 D439A mutation decreased the expression of Miro2. A previous study found that the protein level of Miro in the brains of FAD PS1 mutation patients was decreased, suggesting that Miro may be related to the progression of AD [45]; however, this research did not identify a mechanism linking Miro2 and AD progression. Previous studies have found that Miro overexpression promotes mitochondrial fusion by increasing the expression of the mitochondrial fusion proteins Mfn1/2 [24] and inhibiting the expression of the mitochondrial fission protein Drp1 [11]. The expression levels of Drp1 and Mfn1/2 in fibroblasts and the brains of AD patients were decreased [46]. Our study further found that the expression of Mfn1, Mfn2, and Drp1 was decreased after introduction of the PS2 D439A mutation.

Miro1/2, Mfn1/2, and Drp1 are involved in the maintenance of mitochondrial morphology, size and number and physiological function by regulating mitochondrial fusion and fission dynamics [11, 34]. The increase in the average mitochondrial area may be caused by the increase in swollen mitochondria in PS2 D439A mutant cells (Fig. 6B). Recent studies have shown that Mfn2 knockout (KO) mice exhibit significant dysfunction of mitochondrial fusion/fission dynamics due to a decrease in Mfn2 expression, followed by an increase in the oxidative stress level, which finally leads to the degeneration and death of neurons in the hippocampus and cortex [47]. These pathological changes are indeed the pathological characteristics of AD [34]. In the pyramidal neurons of the CA1 region in 8-week-old Mfn2 KO mice, swollen mitochondria with an increased volume and area and fragmented mitochondrial cristae were observed, and many mitochondria also exhibited vacuolization [47]. Defective mitochondrial cristae (narrow in width and few in number) were observed in PS2-KO cells but not in PS1-KO cells [48]. In our experiments, the TEM results showed that the average length of mitochondria was significantly decreased, suggesting a trend toward increased mitochondrial fission.

Drp1 in mammals is mainly expressed in the cytoplasm, and mitochondrial division depends on the number of Drp1 proteins recruited to the OMM by fission1 and other proteins [49]. Therefore, we further found that the expression of Drp1 in mitochondria was higher in PS2 D439A mutant cells than in PS2 WT cells. Therefore, the balance of mitochondrial fusion/fission in PS2 D439A mutant cells tends to be skewed toward fission. Miro2 and Drp1 play antagonistic roles in the regulation of mitochondrial fusion/fission [21]. Members of a family of proteins containing large GTPase domains play critical roles in enhancing mitochondrial fission (e.g., Drp1) or promoting mitochondrial fusion (e.g., Mfn1 and Mfn2) [50]. A previous study in a mammalian model found physical interactions between Mfn1/Mfn2 and Miro1/Miro2 and observed the strongest interaction between Mfn2 and Miro2 [51]. Miro increases mitochondrial size by inhibiting Drp1 expression under normal cellular conditions; therefore, Miro can mediate mitochondrial fission/fusion dynamics by interacting with other GTPases, such as Mfn1/Mfn2 and Drp1 [11, 52].

Whether the Miro protein can regulate mitochondrial fusion/fission through its interactions with Mfn1/2 in human cells has not been studied. In our study, we found that PS2 failed to bind to Mfn1, Mfn2, or Drp1 in PS2 WT cells, while Miro2 interacted with Mfn1, Mfn2, and Drp1. Moreover, the binding degrees of Miro2 to Mfn1 and Mfn2 in PS2 D439A mutant cells were lower than those in PS2 WT cells. The above results indicated that in addition to regulating mitochondrial fusion/fission by affecting the expression of Mfn1/2 and Drp1, Miro2 may also mediate mitochondrial dynamics by regulating its interaction with Mfn1 or Mfn2, and these results are consistent with previous studies [53].

Miro protein expression was reduced in an AD Drosophila model compared to normal Drosophila. Moreover, the overexpression of Miro in normal Drosophila significantly increased Mfn mRNA expression, and overexpression of Miro in the Drosophila AD model increased the average length of mitochondria [24]. After infection of PS2 D439A mutant SH-SY5Y cells with Ad-Miro2 adenovirus, the increased expression of Miro2 resulted in increases in the Mfn1 and Mfn2 levels but not the Drp1 level compared to those in the Ad-NC group. These results verified that the expression level of Miro2 can affect the dynamics of mitochondrial fusion/fission. Other studies have indicated that the dynamic balance of mitochondrial fission and fusion is shifted toward fission, which may result in the presence of dysfunctional mitochondria in damaged neurons [50].

Ultrastructural morphometric analysis revealed that mitochondria have significant structural damage, such as fragmented cristae or nearly total loss of the inner structure, which probably contributes to mitochondrial dysfunction and increased ROS levels in the AD brain [53]. In the AD brain, the mitochondrial size was also observed to be significantly increased and the mitochondrial number decreased [53]. In AD neurons, a significantly reduced mitochondrial length but increased width were observed, and the overall mitochondrial size was significantly increased [54]. Our TEM results in PS2 D439A mutant SH-SY5Y cells confirmed the above observations.

The maintenance of normal mitochondrial function depends on an intact mitochondrial structure, and increased production of mitochondrial ROS, impaired mitochondrial function, and apoptosis are related to an abnormal internal structure of mitochondria and an imbalance in the fission/fusion machinery [55, 56]. In PS2 D439A mutant cells, the MMP was significantly lower than that in PS2 WT cells, which indicated that mitochondrial function was impaired in PS2 D439A mutant cells. Mitochondrial fission or fusion deficiency may reduce the MMP [25]. Maintenance of the membrane potential of the inner mitochondrial membrane (IMM) is necessary for mitochondrial fusion [56]. In PS2^−/−^ MEFs, the MMP was significantly decreased, indicating that the deletion of PS2 significantly increased the proportion of nonfunctional mitochondria, which suggests that the deletion of PS2 is directly associated with deleterious mitochondrial changes [57].

In AD, mitochondrial dysfunction has been considered the main cause of ROS production; reciprocally, mitochondria are the main target of oxidative damage [40]. In our study, the level of ROS was significantly increased in PS2 D439A mutant cells. More than 90% of ROS are generated in mitochondria, and excessive mitochondrial fragmentation causes increased ROS production [58]. Oxidative stress further leads to glycolysis inhibition and mitochondrial dysfunction [59]. In PS2 D439A mutant cells, the decrease in mitochondrial length and the structural damage to mitochondrial cristae indicated excessive fragmentation of mitochondria, which may be the main source of ROS generation. Oxidative stress continually reduces GSH-Px and SOD2 levels, while increasing MDA levels [26, 60]. SOD2, existing in mitochondrial spaces, is known to be one of the most important endogenous antioxidants [60]. We found that PS2 D439A mutant cells had higher MDA levels but lower SOD2 and GSH-Px levels than PS2 WT cells (Fig. 8E, F, G), suggesting the presence of an imbalance in oxidant related parameters after introduction of the PS2 D439A mutation. In AD, increased ROS production can lead to the accumulation of Aβ protein, eventually causing neuronal death [61]. Increased production of free radicals and lipid peroxidation preceded the formation of Aβ plaques in an AD animal model [62]. Mitochondrial Aβ production is regulated by mitochondrial biogenesis, suggesting that mitochondrial dysfunction is an upstream event in the pathological progression of AD [63]. In addition, the ATP level was decreased markedly in the PS2 D439A mutant cells (Fig. 8H). Mitochondrial dysfunction was correlated with the concentration of intracellular ATP and the energy balance of the cell [64].

Mitochondrial oxidative stress further promotes the opening of the mitochondrial permeability transition pore (MPTP), which triggers the release of Cyt c from mitochondria into the cytosol, leading to a decrease in the MMP and to ROS burst, thus forming a vicious cycle and further aggravating mitochondrial damage and cell apoptosis [60, 63]. Key molecules in the apoptotic pathway include the BCL-2 family and caspase cysteine protease family proteins, and the overexpression of the FAD-associated mutant PS2 N141I was found to increase the sensitivity of neurons to apoptotic stimulation compared with PS2 WT overexpression; in addition, neurons expressing the PS2 N141I mutant exhibited a reduced BCL-2 expression level [65]. Our results showed that the PS2 D439A mutant significantly decreased the BCL-2 protein level compared with that in PS2 WT cells. The BCL-2/BAX ratio was decreased in PS2 D439A mutant cells, indicating that the PS2 D439A mutation affects the balance of apoptosis regulation. BCL-2 and mitochondrial fission and fusion proteins may form a regulatory network whose function is to sense the health status of cells [62]. Antiapoptotic BCL-2 family members such as BCL-2 promote cell survival by preventing BAX from multimerizing on the mitochondrial surface to allow the release of Cyt c [66]. Therefore, downregulation of BCL-2 expression would be expected to promote the release of Cyt c from mitochondria into the cytosol. Indeed, our results showed that the Cyt c level was significantly increased in the cytosol and was decreased in mitochondria in PS2 D439A mutant cells compared with PS2 WT cells. This observation explains the decrease in the viability of PS2 D439A mutant cells. Moreover, the release of Cyt C from mitochondria occurs before caspase activation [66].

The amyloid cascade hypothesis does not easily account for various parameters associated with AD [67], while the primary mitochondrial cascade hypothesis assumes mitochondrial pathology hierarchically supersedes Aβ pathology [6]. The debate about the origin of mitochondrial changes in AD continues, and some argue that Aβ induces mitochondrial dysfunction in AD [6]. Membrane-associated oxidative stress, mitochondrial alterations and apoptosis are present during Aβ-mediated neuronal degeneration during the AD process [68]. Hence, the possibility of a feedback loop between mitochondrial pathology and Aβ pathology cannot be eliminated. A previous study found that the PS2 D439A mutation did not change the Aβ42/40 ratio or Aβ42 level [16]. We found that the percentage of apoptotic cells in the PS2 D439A mutant group was significantly higher than that in the PS2 WT group in the AD cell model induced by Aβ25-35. Although mitochondrial dysfunction precedes Aβ formation, once Aβ penetrates the cell membrane after aggregation, it further exacerbates the decline in mitochondrial function [53]. Previous research results indicated that the increased production of Aβ and the interaction of Aβ with Drp1 are crucial factors in mitochondrial fragmentation and the imbalance in mitochondrial dynamics in patients with AD [69]. Therefore, increased Aβ deposition may further lead to mitochondrial dysfunction and then increase apoptosis in PS2 D439A-mutant SH-SY5Y cells.

Taken together, our results indicate that alterations in the interaction between PS2 and Miro2, decreased Miro2 expression and GTPase activity and dysfunction of mitochondrial fission/fusion dynamics in the setting of the PS2 D439A mutation may have a profound impact on the pathogenesis of AD (Supplementary Fig. 4). However, the extrapolation of the present results of a single in vitro cell-level experiment to the pathogenesis of AD appears premature. Therefore, further studies are needed at the animal level to verify the pathogenic role of the imbalance in mitochondrial dynamics caused by the PS2 D439A mutation in the pathogenesis of AD.

## Conclusions

In summary, the results from RNA-sequencing analysis suggest that *PS2* gene knockdown may participate in the pathogenesis of AD by affecting the regulation of GTPase activity. Additionally, our study found for the first time that PS2 can bind to Miro2, which is a key player in mitochondrial dynamics in AD, and the PS2 D439A mutation weakens the interaction between PS2 and Miro2 and decreases the expression of Miro2, Mfn1, and Mfn2 and the GTPase activity of Miro2, while the number of Drp1 molecules localized on the OMM increases, which leads to dysfunction of mitochondrial fusion and fission dynamics and changes in mitochondrial morphology. These changes result in increased oxidative stress and apoptosis, which are closely related to the pathogenesis of AD. Thus, our findings imply that the PS2 D439A mutation may be a major trigger for the imbalance of mitochondrial fusion/fission dynamics, offering new molecular insights into the “primary mitochondrial cascade hypothesis” of AD pathogenesis. With these insights, potential therapeutic strategies for AD can be developed.

### Supplementary Information

Below is the link to the electronic supplementary material.

Supplementary file1 (DOCX 2097 KB)

## Data Availability

The data that support the findings of this study are openly available in NCBI SRA (BioProject ID: PRJNA963881): https://www.ncbi.nlm.nih.gov/bioproject/PRJNA963881. Further inquiries can be directed to the corresponding author.

## Abbreviations

AD: Alzheimer’s disease
Aβ: Amyloid-beta
APP: Amyloid precursor protein
APPsw: APP with Swedish mutation
ARHGEF5: Rho guanine nucleotide exchange factor 5
CCK8: Cell counting kit-8
Co-IP: Co-immunoprecipitation
DCFH-DA: 2′,7′-Dichlorodihydro fluorescein diacetate
DEGs: Differential expression genes
DMEM: Dulbecco’s modified Eagle’s medium
DO: Disease Ontology
DPBS: Dulbecco’s phosphate-buffered saline
EOFAD: Early-onset familial Alzheimer’s disease
FAD: Familial Alzheimer’s disease
FBS: Fetal bovine serum
FDR: False discovery rate
FPKM: Fragment per kilobase of transcript per million mapped reads
GABA: Gamma-aminobutyric acid
GSH-Px: Glutathione peroxidase
GNG3: G protein subunit gamma 3
GO: Gene Ontology
GST: Glutathione S-transferase
JUN: Jun proto-oncogene/AP-1 transcription factor subunit
KEGG: Kyoto Encyclopedia of Genes and Genomes
PS: Presenilin
qRT-PCR: Quantitative real-time PCR
TEM: Transmission electron microscopy
MDA: Malondialdehyde (MDA)
MEFs: Mouse embryonic fibroblasts
Mfn: Mitofusin
MMP: Mitochondrial membrane potential
MOI: Multiplicity of infection
NFTs: Neurofibrillary tangles
GAPs: GTPase-activating proteins
GEF: Guanine nucleotide exchange factors
OMM: Outer mitochondrial membrane
PLXNA4: Plexin A4
RAPGEF5: Rap guanine nucleotide exchange factor 5
RELN: Reelin
RGPD8: Glutamate receptor interaction domain 8
ROS: Reactive oxygen species
SFRP1: Secretory frizzled related protein 1
SPRY1: Sprout receptor tyrosine kinase signal antagonist 1
SOD2: Human superoxide dismutase 2
WT: Wild type
