# Supplementary material for: Presenilin2 D439A Mutation Induces Dysfunction of Mitochondrial Fusion/Fission Dynamics and Abnormal Regulation of GTPase Activity
Source: Mol Neurobiol. 2023 Dec 30;61(8):5047–70. doi: 10.1007/s12035-023-03858-y (PMC11249618; doi:10.1007/s12035-023-03858-y)
Supplement: Supplementary file 1 — Supplementary file1 (DOCX 2097 KB) [file 12035_2023_3858_MOESM1_ESM.docx]

Supplementary Material

**Presenilin2 D439A mutation induces dysfunction of mitochondrial fusion/fission dynamics and abnormal regulation of GTPase activity**

Chenhao Gao**^1,2#^** · **Junkui Shang^1,3#^** · **Zhengyu Sun ^1,3^** · **Mingrong Xia^1^** · **Dandan Gao^1^** · **Ruihua Sun^1,2^** · **Wei Li^1^** · **Fengyu Wang^1,3^** · **Jiewen Zhang^1,2,3^***

^1^ Department of Neurology, Zhengzhou University People’s Hospital, Henan Provincial People’s Hospital, Zhengzhou, Henan, 450003, China.

^2^ Academy of Medical Sciences, Zhengzhou University, Zhengzhou, Henan, 450003, China.

^3^ Department of Neurology, Henan University People’s Hospital, Henan Provincial People’s Hospital, Zhengzhou, Henan, 450003, China.

*Corresponding authors: zhangjiewen9900@126.com

^#^ These authors have contributed equally to this work.

## Supplementary Figures


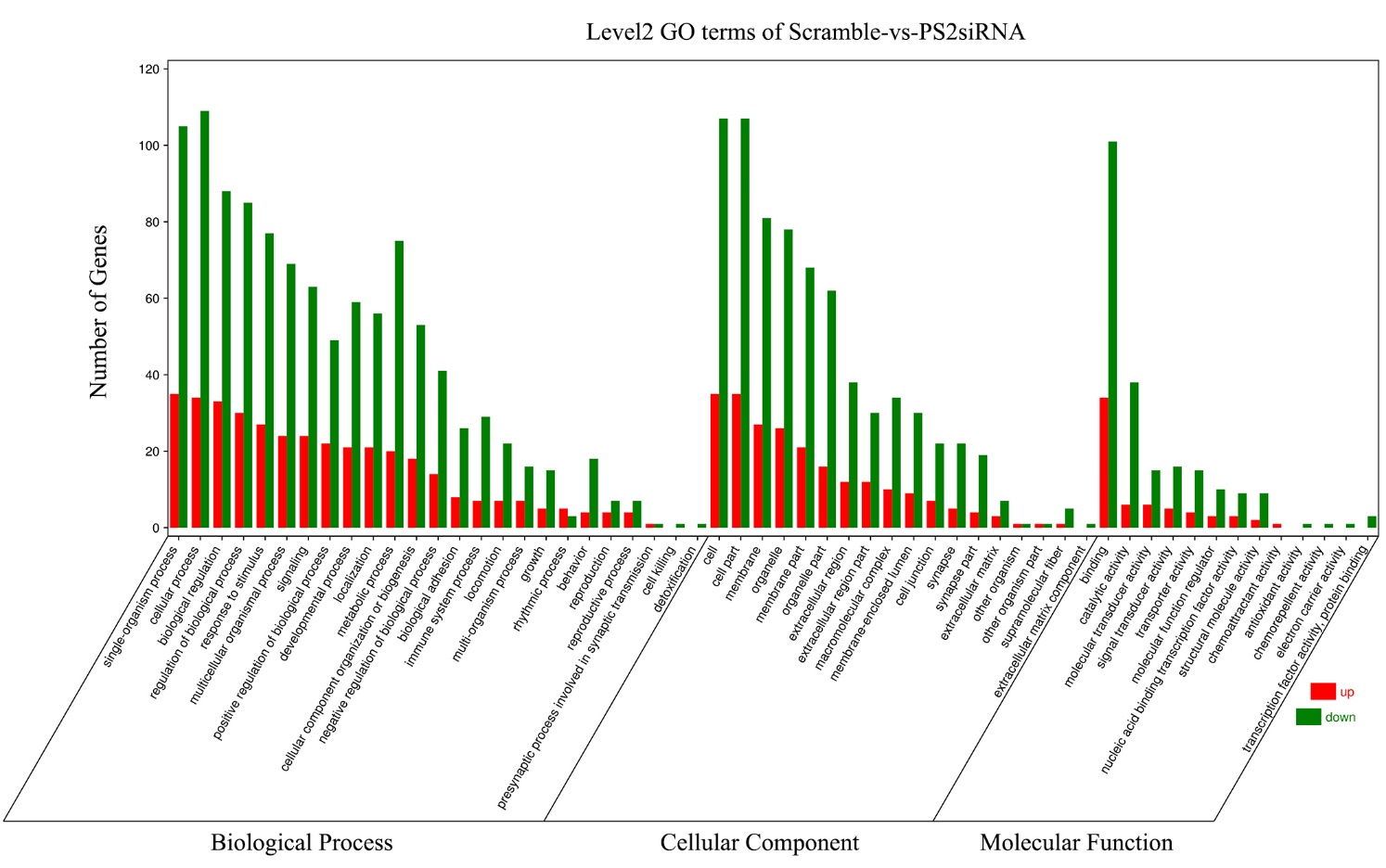
**Supplementary Fig. 1** The GO enrichment classification histogram of differential genes in scramble and PS2 siRNA groups. The figure shows the GO enrichment results, where the X axis is the GO entry and the Y axis is the number of genes. Red represents gene up regulation and green represents gene down regulation.


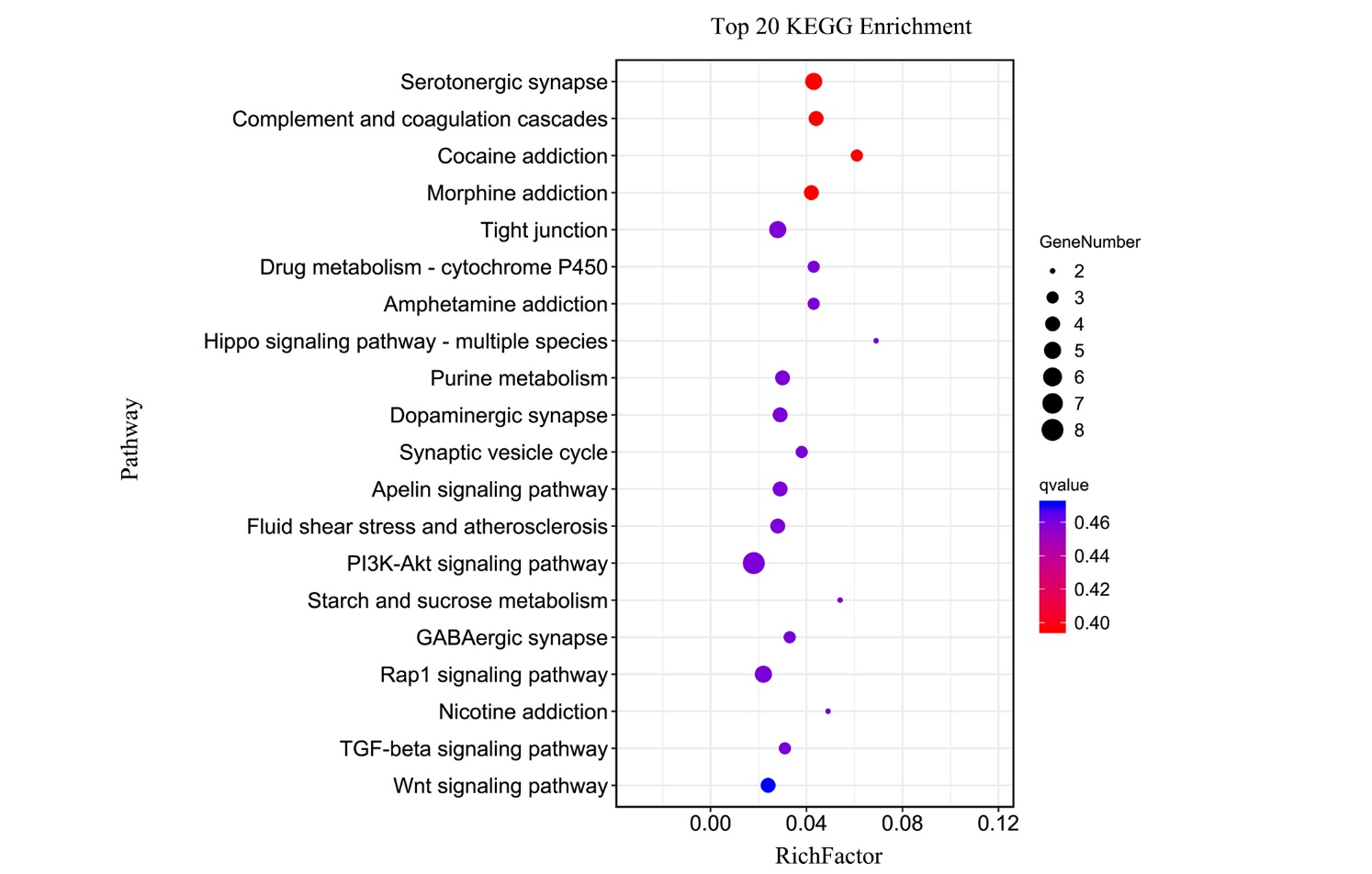


**Supplementary Fig. 2** KEGG enrichment Bubble Diagram. The vertical coordinate is the pathway, and the horizontal coordinate is the Rich factor (the difference genes in the path are divided by all the numbers). The bubble size is proportional to the number of genes. The red color means the smaller the Q value.


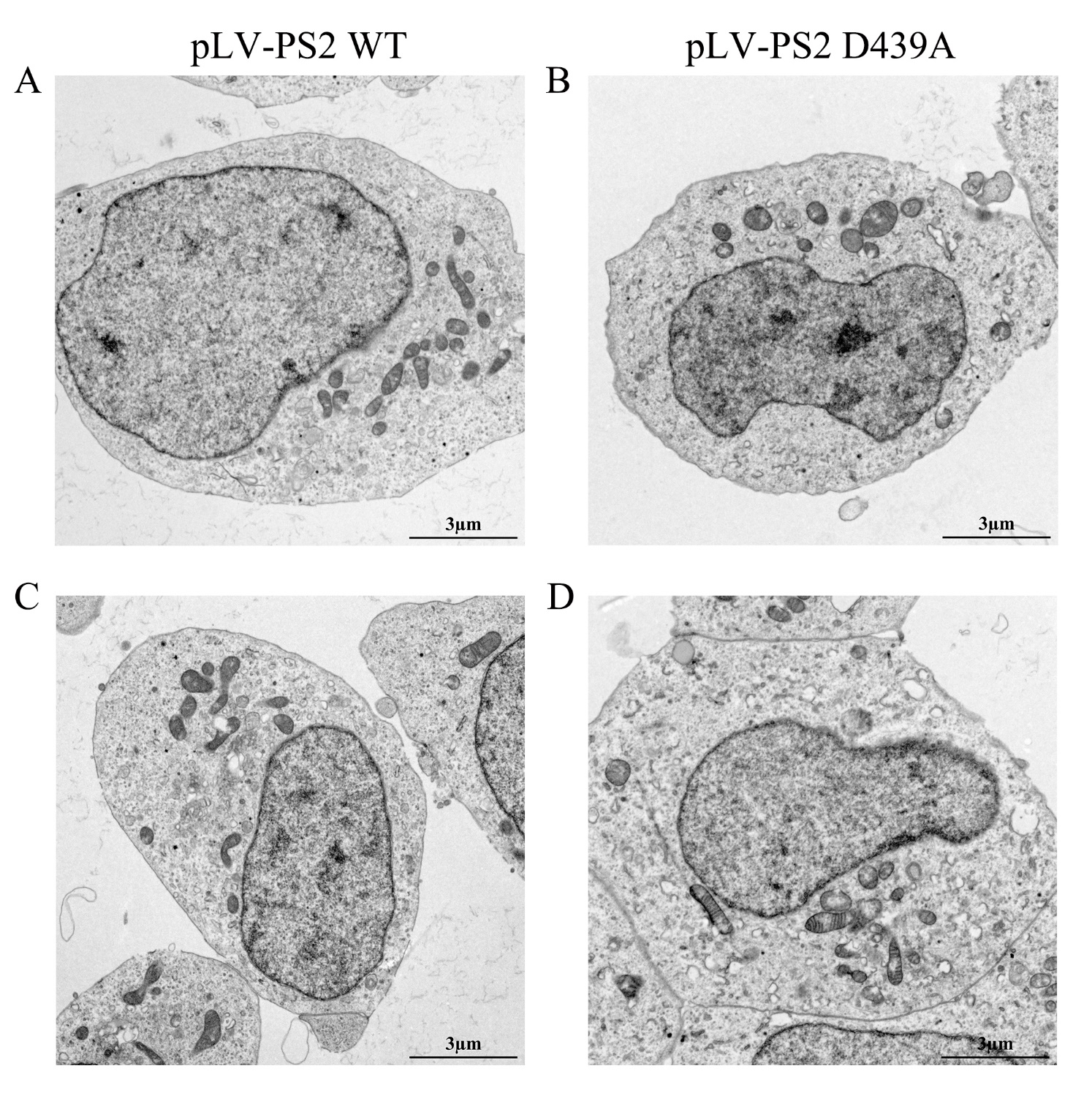


**Supplementary Fig. 3**. TEM of mitochondria in SH-SY5Y cells transduced with pLV-PS2 WT(**A，C**) and pLV-PS2 D439A mutant(**B，D**). Scale bars are indicated on the bottom of the pictures.


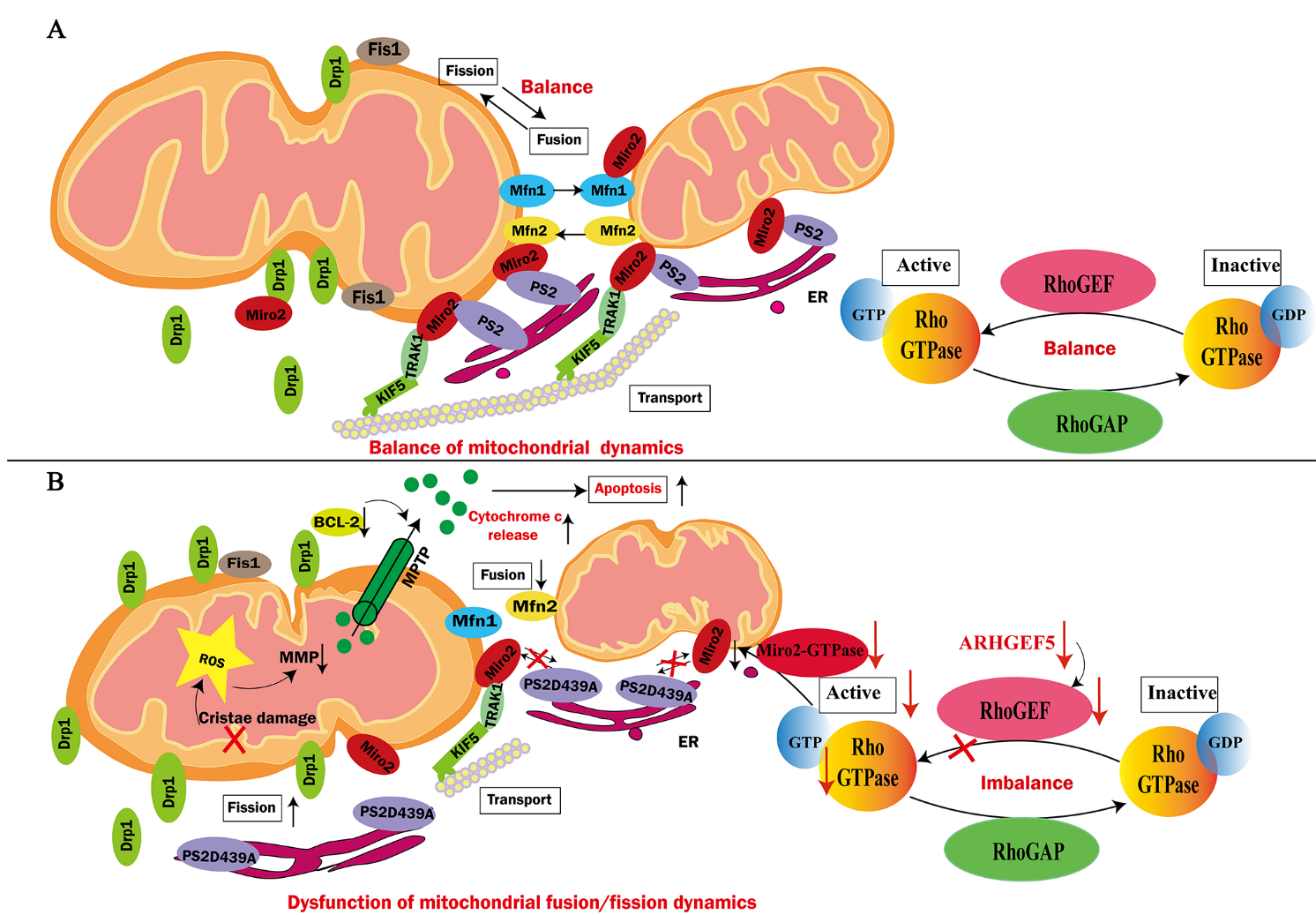


**Supplementary Fig. 4** Schematic representation of the PS2 D439A mutation induced dysfunction of mitochondrial fusion/fission dynamics. **A** Mitochondria are dynamic organelles undergoing coordinated cycles of fusion, fission and transport. Mitochondrial fusion is promoted by the formation of Mfn1/Mfn2 homotypic or heterotypic complexes, while mitochondrial fission is mainly regulated by Fis1 and Drp1. PS2 can interact with Miro2. Miro mediates mitochondrial fission/fusion dynamics by interacting with Mfn1/Mfn2 (Mfn2 interacts with Miro2 more strongly than with Miro1) and Drp1 proteins and then increasing the expression of Mfn1 and Mfn2 to induce mitochondrial fusion. Miro regulates mitochondrial anterograde and retrograde transport based on microtubules. These dynamic processes control the size, number and morphology of mitochondria, thus controlling mitochondrial function, distribution and transport. These biological processes are mediated by several GTPases related to mitochondrial dynamics. *ARHGEF5* plays an important role in the regulation of endogenous Rho GTPases. **B** The PS2 D439A mutation may lead to a decrease in Miro2-GTPase activity by regulating the decrease in *ARHGEF5* expression, which may then affect the balance of mitochondrial dynamics. The PS2 D439A mutation weakens the interaction between PS2 and Miro2 and decreases the expression of Miro2, Mfn1 and Mfn2, while the number of Drp1 molecules localized on the OMM increases, which leads to dysfunction of mitochondrial fusion and fission dynamics and changes in mitochondrial morphology (decreased mitochondrial length and fragmented mitochondrial cristae). These changes can increase the production of mitochondrial ROS and then decrease the MPP. Increased ROS triggers the release of Cyt c from mitochondria into the cytoplasm through MPTP opening, and the PS2 D439A mutation reduces the BCL-2 protein level, further inducing MPTP opening and promoting the activation of apoptosis.
